# Supplementary material for: Potential Role of the mTORC1-PGC1α-PPARα Axis under Type-II Diabetes and Hypertension in the Human Heart
Source: Int J Mol Sci. 2023 May 11;24(10):8629. doi: 10.3390/ijms24108629 (PMC10218005; doi:10.3390/ijms24108629)
Supplement: Supplementary file 1 [file ijms-24-08629-s001.zip › ijms-2338464-supplementary.pptx]

## Slide 1
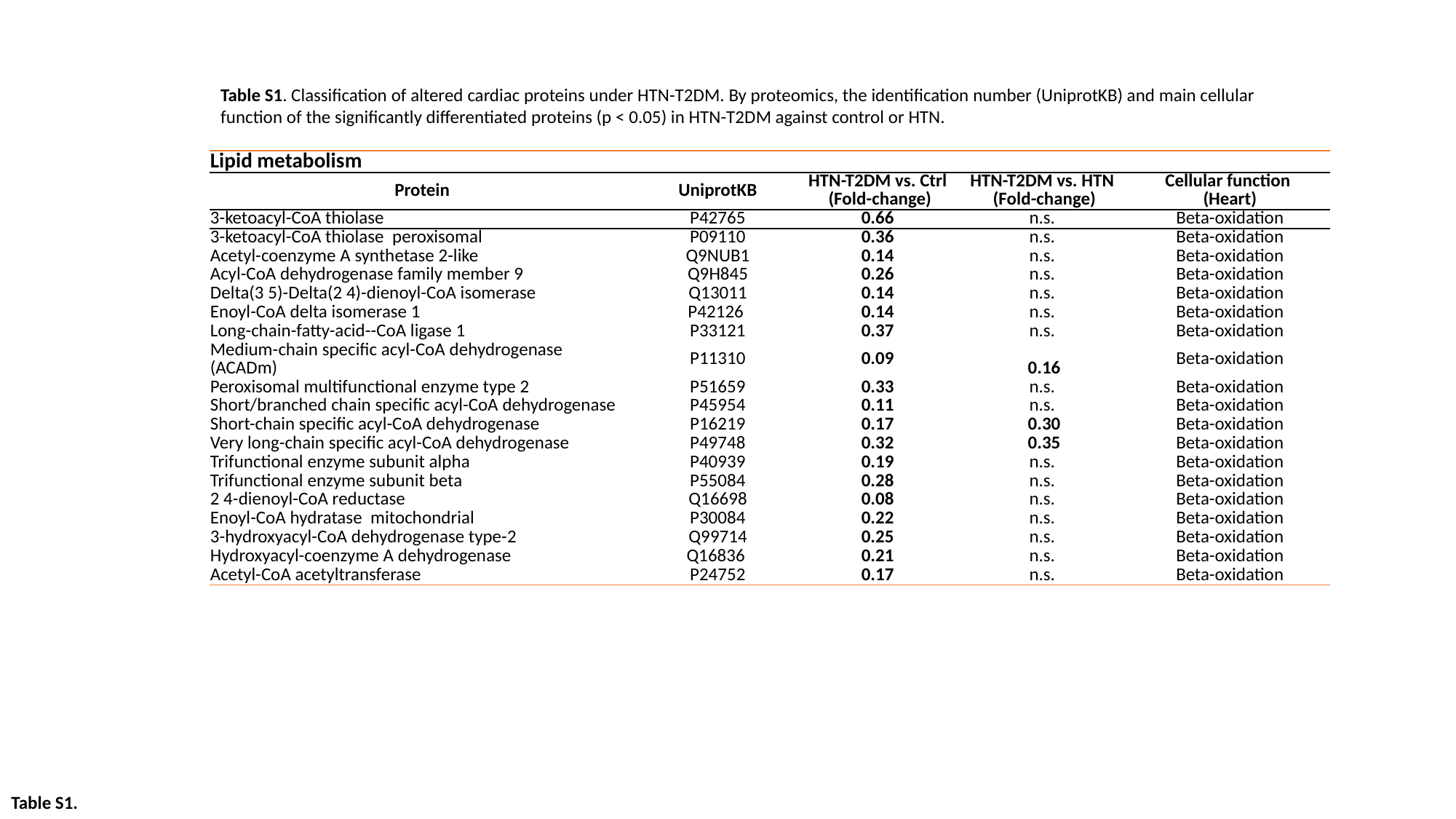

Table S1. Classification of altered cardiac proteins under HTN-T2DM. By proteomics, the identification number (UniprotKB) and main cellular function of the significantly differentiated proteins (p < 0.05) in HTN-T2DM against control or HTN.
| Lipid metabolism | | | | |
| --- | --- | --- | --- | --- |
| Protein | UniprotKB | HTN-T2DM vs. Ctrl (Fold-change) | HTN-T2DM vs. HTN (Fold-change) | Cellular function (Heart) |
| 3-ketoacyl-CoA thiolase | P42765 | 0.66 | n.s. | Beta-oxidation |
| 3-ketoacyl-CoA thiolase peroxisomal | P09110 | 0.36 | n.s. | Beta-oxidation |
| Acetyl-coenzyme A synthetase 2-like | Q9NUB1 | 0.14 | n.s. | Beta-oxidation |
| Acyl-CoA dehydrogenase family member 9 | Q9H845 | 0.26 | n.s. | Beta-oxidation |
| Delta(3 5)-Delta(2 4)-dienoyl-CoA isomerase | Q13011 | 0.14 | n.s. | Beta-oxidation |
| Enoyl-CoA delta isomerase 1 | P42126 | 0.14 | n.s. | Beta-oxidation |
| Long-chain-fatty-acid--CoA ligase 1 | P33121 | 0.37 | n.s. | Beta-oxidation |
| Medium-chain specific acyl-CoA dehydrogenase (ACADm) | P11310 | 0.09 | 0.16 | Beta-oxidation |
| Peroxisomal multifunctional enzyme type 2 | P51659 | 0.33 | n.s. | Beta-oxidation |
| Short/branched chain specific acyl-CoA dehydrogenase | P45954 | 0.11 | n.s. | Beta-oxidation |
| Short-chain specific acyl-CoA dehydrogenase | P16219 | 0.17 | 0.30 | Beta-oxidation |
| Very long-chain specific acyl-CoA dehydrogenase | P49748 | 0.32 | 0.35 | Beta-oxidation |
| Trifunctional enzyme subunit alpha | P40939 | 0.19 | n.s. | Beta-oxidation |
| Trifunctional enzyme subunit beta | P55084 | 0.28 | n.s. | Beta-oxidation |
| 2 4-dienoyl-CoA reductase | Q16698 | 0.08 | n.s. | Beta-oxidation |
| Enoyl-CoA hydratase mitochondrial | P30084 | 0.22 | n.s. | Beta-oxidation |
| 3-hydroxyacyl-CoA dehydrogenase type-2 | Q99714 | 0.25 | n.s. | Beta-oxidation |
| Hydroxyacyl-coenzyme A dehydrogenase | Q16836 | 0.21 | n.s. | Beta-oxidation |
| Acetyl-CoA acetyltransferase | P24752 | 0.17 | n.s. | Beta-oxidation |
Table S1.

## Slide 2
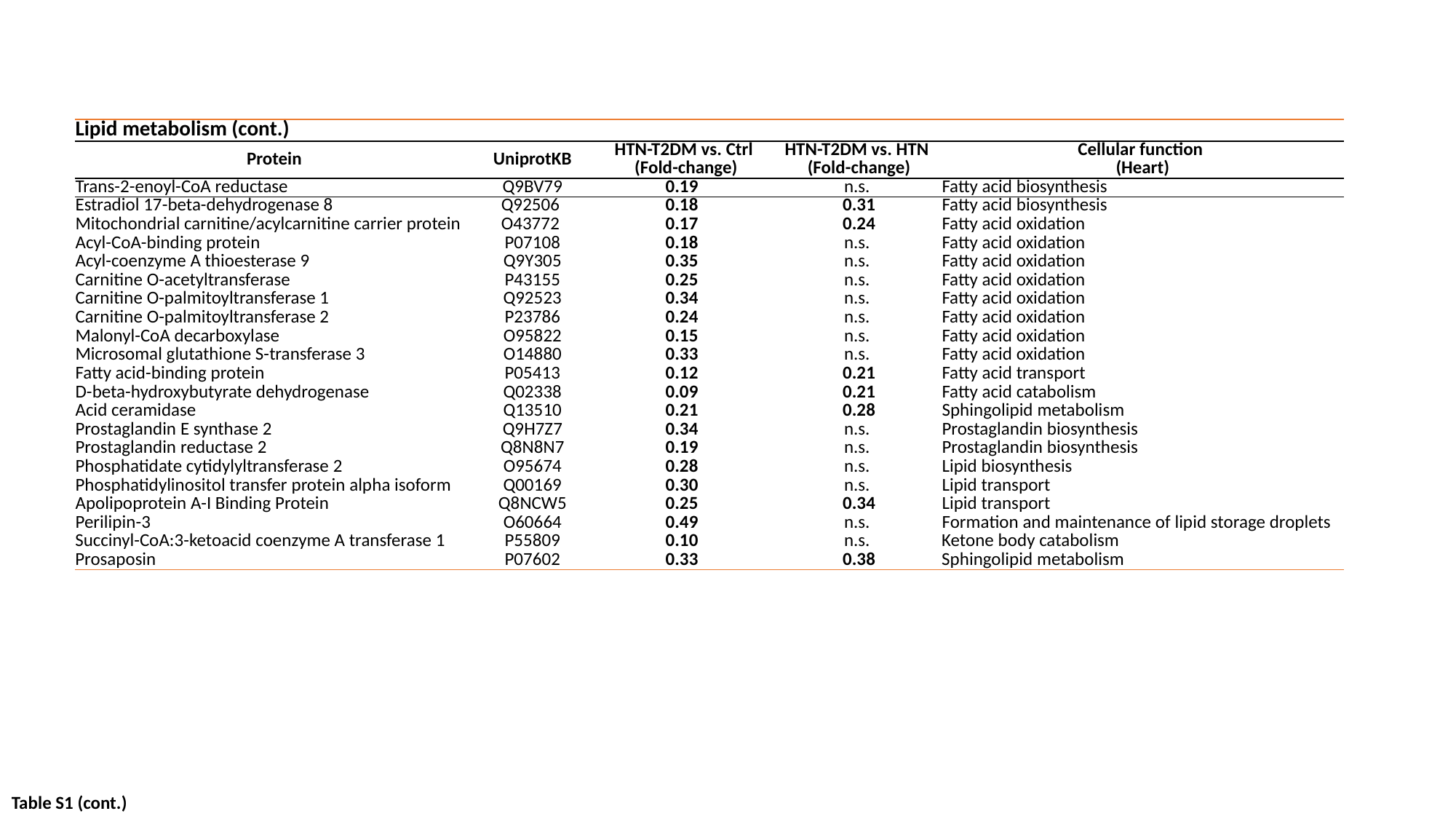

| Lipid metabolism (cont.) | | | | |
| --- | --- | --- | --- | --- |
| Protein | UniprotKB | HTN-T2DM vs. Ctrl (Fold-change) | HTN-T2DM vs. HTN (Fold-change) | Cellular function (Heart) |
| Trans-2-enoyl-CoA reductase | Q9BV79 | 0.19 | n.s. | Fatty acid biosynthesis |
| Estradiol 17-beta-dehydrogenase 8 | Q92506 | 0.18 | 0.31 | Fatty acid biosynthesis |
| Mitochondrial carnitine/acylcarnitine carrier protein | O43772 | 0.17 | 0.24 | Fatty acid oxidation |
| Acyl-CoA-binding protein | P07108 | 0.18 | n.s. | Fatty acid oxidation |
| Acyl-coenzyme A thioesterase 9 | Q9Y305 | 0.35 | n.s. | Fatty acid oxidation |
| Carnitine O-acetyltransferase | P43155 | 0.25 | n.s. | Fatty acid oxidation |
| Carnitine O-palmitoyltransferase 1 | Q92523 | 0.34 | n.s. | Fatty acid oxidation |
| Carnitine O-palmitoyltransferase 2 | P23786 | 0.24 | n.s. | Fatty acid oxidation |
| Malonyl-CoA decarboxylase | O95822 | 0.15 | n.s. | Fatty acid oxidation |
| Microsomal glutathione S-transferase 3 | O14880 | 0.33 | n.s. | Fatty acid oxidation |
| Fatty acid-binding protein | P05413 | 0.12 | 0.21 | Fatty acid transport |
| D-beta-hydroxybutyrate dehydrogenase | Q02338 | 0.09 | 0.21 | Fatty acid catabolism |
| Acid ceramidase | Q13510 | 0.21 | 0.28 | Sphingolipid metabolism |
| Prostaglandin E synthase 2 | Q9H7Z7 | 0.34 | n.s. | Prostaglandin biosynthesis |
| Prostaglandin reductase 2 | Q8N8N7 | 0.19 | n.s. | Prostaglandin biosynthesis |
| Phosphatidate cytidylyltransferase 2 | O95674 | 0.28 | n.s. | Lipid biosynthesis |
| Phosphatidylinositol transfer protein alpha isoform | Q00169 | 0.30 | n.s. | Lipid transport |
| Apolipoprotein A-I Binding Protein | Q8NCW5 | 0.25 | 0.34 | Lipid transport |
| Perilipin-3 | O60664 | 0.49 | n.s. | Formation and maintenance of lipid storage droplets |
| Succinyl-CoA:3-ketoacid coenzyme A transferase 1 | P55809 | 0.10 | n.s. | Ketone body catabolism |
| Prosaposin | P07602 | 0.33 | 0.38 | Sphingolipid metabolism |
Table S1 (cont.)

## Slide 3
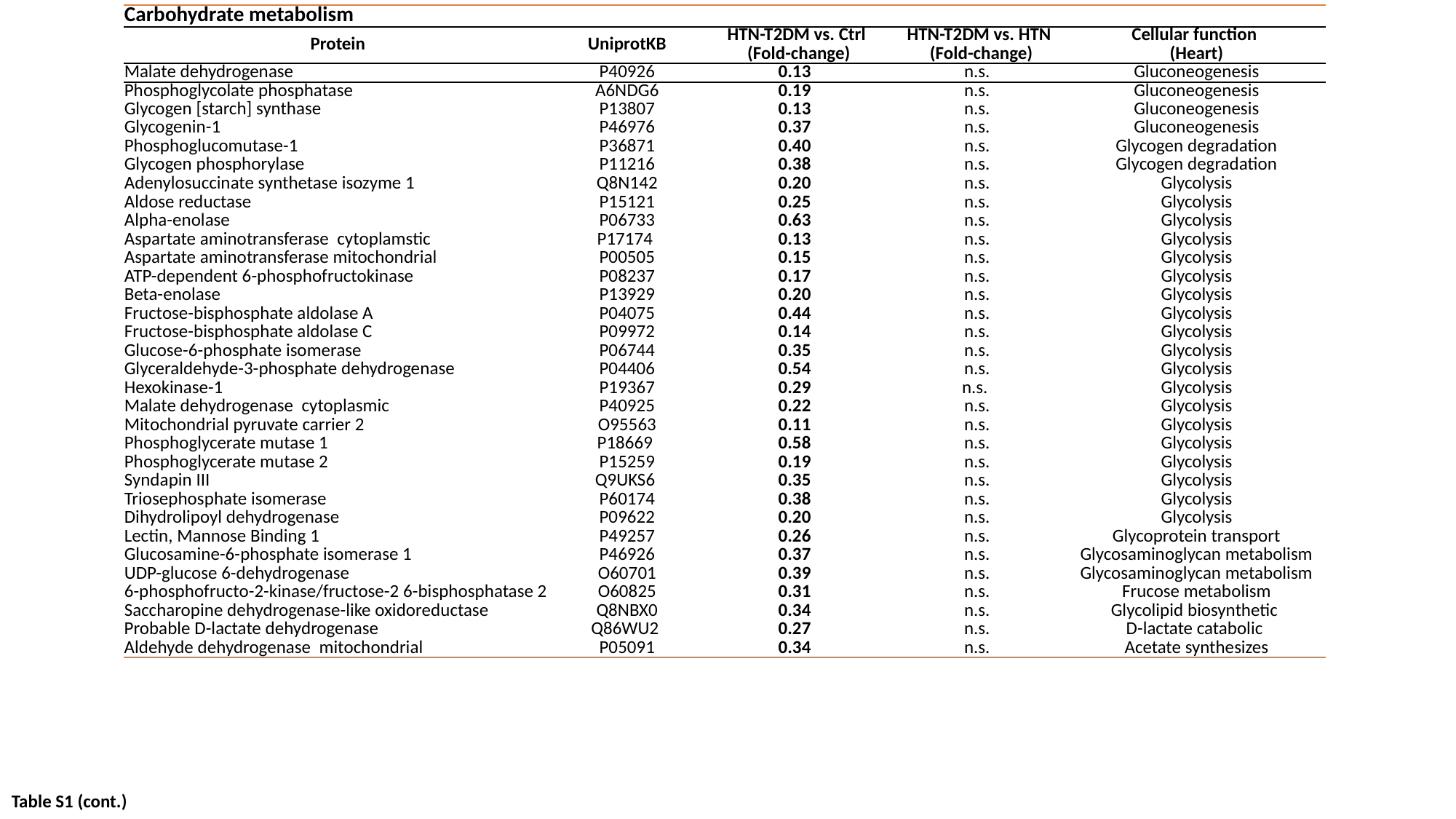

| Carbohydrate metabolism | | | | |
| --- | --- | --- | --- | --- |
| Protein | UniprotKB | HTN-T2DM vs. Ctrl (Fold-change) | HTN-T2DM vs. HTN (Fold-change) | Cellular function (Heart) |
| Malate dehydrogenase | P40926 | 0.13 | n.s. | Gluconeogenesis |
| Phosphoglycolate phosphatase | A6NDG6 | 0.19 | n.s. | Gluconeogenesis |
| Glycogen [starch] synthase | P13807 | 0.13 | n.s. | Gluconeogenesis |
| Glycogenin-1 | P46976 | 0.37 | n.s. | Gluconeogenesis |
| Phosphoglucomutase-1 | P36871 | 0.40 | n.s. | Glycogen degradation |
| Glycogen phosphorylase | P11216 | 0.38 | n.s. | Glycogen degradation |
| Adenylosuccinate synthetase isozyme 1 | Q8N142 | 0.20 | n.s. | Glycolysis |
| Aldose reductase | P15121 | 0.25 | n.s. | Glycolysis |
| Alpha-enolase | P06733 | 0.63 | n.s. | Glycolysis |
| Aspartate aminotransferase cytoplamstic | P17174 | 0.13 | n.s. | Glycolysis |
| Aspartate aminotransferase mitochondrial | P00505 | 0.15 | n.s. | Glycolysis |
| ATP-dependent 6-phosphofructokinase | P08237 | 0.17 | n.s. | Glycolysis |
| Beta-enolase | P13929 | 0.20 | n.s. | Glycolysis |
| Fructose-bisphosphate aldolase A | P04075 | 0.44 | n.s. | Glycolysis |
| Fructose-bisphosphate aldolase C | P09972 | 0.14 | n.s. | Glycolysis |
| Glucose-6-phosphate isomerase | P06744 | 0.35 | n.s. | Glycolysis |
| Glyceraldehyde-3-phosphate dehydrogenase | P04406 | 0.54 | n.s. | Glycolysis |
| Hexokinase-1 | P19367 | 0.29 | n.s. | Glycolysis |
| Malate dehydrogenase cytoplasmic | P40925 | 0.22 | n.s. | Glycolysis |
| Mitochondrial pyruvate carrier 2 | O95563 | 0.11 | n.s. | Glycolysis |
| Phosphoglycerate mutase 1 | P18669 | 0.58 | n.s. | Glycolysis |
| Phosphoglycerate mutase 2 | P15259 | 0.19 | n.s. | Glycolysis |
| Syndapin III | Q9UKS6 | 0.35 | n.s. | Glycolysis |
| Triosephosphate isomerase | P60174 | 0.38 | n.s. | Glycolysis |
| Dihydrolipoyl dehydrogenase | P09622 | 0.20 | n.s. | Glycolysis |
| Lectin, Mannose Binding 1 | P49257 | 0.26 | n.s. | Glycoprotein transport |
| Glucosamine-6-phosphate isomerase 1 | P46926 | 0.37 | n.s. | Glycosaminoglycan metabolism |
| UDP-glucose 6-dehydrogenase | O60701 | 0.39 | n.s. | Glycosaminoglycan metabolism |
| 6-phosphofructo-2-kinase/fructose-2 6-bisphosphatase 2 | O60825 | 0.31 | n.s. | Frucose metabolism |
| Saccharopine dehydrogenase-like oxidoreductase | Q8NBX0 | 0.34 | n.s. | Glycolipid biosynthetic |
| Probable D-lactate dehydrogenase | Q86WU2 | 0.27 | n.s. | D-lactate catabolic |
| Aldehyde dehydrogenase mitochondrial | P05091 | 0.34 | n.s. | Acetate synthesizes |
Table S1 (cont.)

## Slide 4
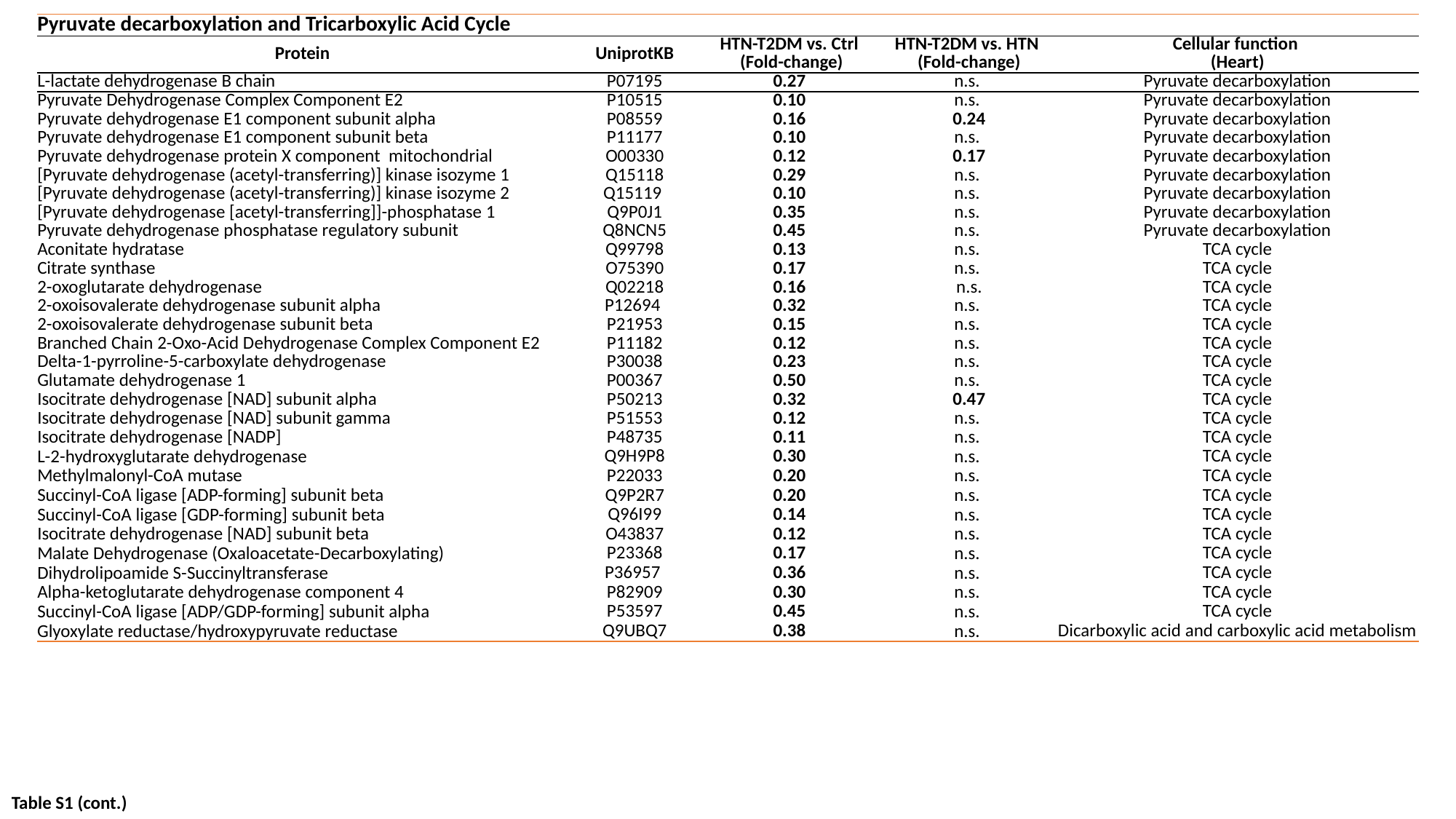

| Pyruvate decarboxylation and Tricarboxylic Acid Cycle | | | | |
| --- | --- | --- | --- | --- |
| Protein | UniprotKB | HTN-T2DM vs. Ctrl (Fold-change) | HTN-T2DM vs. HTN (Fold-change) | Cellular function (Heart) |
| L-lactate dehydrogenase B chain | P07195 | 0.27 | n.s. | Pyruvate decarboxylation |
| Pyruvate Dehydrogenase Complex Component E2 | P10515 | 0.10 | n.s. | Pyruvate decarboxylation |
| Pyruvate dehydrogenase E1 component subunit alpha | P08559 | 0.16 | 0.24 | Pyruvate decarboxylation |
| Pyruvate dehydrogenase E1 component subunit beta | P11177 | 0.10 | n.s. | Pyruvate decarboxylation |
| Pyruvate dehydrogenase protein X component mitochondrial | O00330 | 0.12 | 0.17 | Pyruvate decarboxylation |
| [Pyruvate dehydrogenase (acetyl-transferring)] kinase isozyme 1 | Q15118 | 0.29 | n.s. | Pyruvate decarboxylation |
| [Pyruvate dehydrogenase (acetyl-transferring)] kinase isozyme 2 | Q15119 | 0.10 | n.s. | Pyruvate decarboxylation |
| [Pyruvate dehydrogenase [acetyl-transferring]]-phosphatase 1 | Q9P0J1 | 0.35 | n.s. | Pyruvate decarboxylation |
| Pyruvate dehydrogenase phosphatase regulatory subunit | Q8NCN5 | 0.45 | n.s. | Pyruvate decarboxylation |
| Aconitate hydratase | Q99798 | 0.13 | n.s. | TCA cycle |
| Citrate synthase | O75390 | 0.17 | n.s. | TCA cycle |
| 2-oxoglutarate dehydrogenase | Q02218 | 0.16 | n.s. | TCA cycle |
| 2-oxoisovalerate dehydrogenase subunit alpha | P12694 | 0.32 | n.s. | TCA cycle |
| 2-oxoisovalerate dehydrogenase subunit beta | P21953 | 0.15 | n.s. | TCA cycle |
| Branched Chain 2-Oxo-Acid Dehydrogenase Complex Component E2 | P11182 | 0.12 | n.s. | TCA cycle |
| Delta-1-pyrroline-5-carboxylate dehydrogenase | P30038 | 0.23 | n.s. | TCA cycle |
| Glutamate dehydrogenase 1 | P00367 | 0.50 | n.s. | TCA cycle |
| Isocitrate dehydrogenase [NAD] subunit alpha | P50213 | 0.32 | 0.47 | TCA cycle |
| Isocitrate dehydrogenase [NAD] subunit gamma | P51553 | 0.12 | n.s. | TCA cycle |
| Isocitrate dehydrogenase [NADP] | P48735 | 0.11 | n.s. | TCA cycle |
| L-2-hydroxyglutarate dehydrogenase | Q9H9P8 | 0.30 | n.s. | TCA cycle |
| Methylmalonyl-CoA mutase | P22033 | 0.20 | n.s. | TCA cycle |
| Succinyl-CoA ligase [ADP-forming] subunit beta | Q9P2R7 | 0.20 | n.s. | TCA cycle |
| Succinyl-CoA ligase [GDP-forming] subunit beta | Q96I99 | 0.14 | n.s. | TCA cycle |
| Isocitrate dehydrogenase [NAD] subunit beta | O43837 | 0.12 | n.s. | TCA cycle |
| Malate Dehydrogenase (Oxaloacetate-Decarboxylating) | P23368 | 0.17 | n.s. | TCA cycle |
| Dihydrolipoamide S-Succinyltransferase | P36957 | 0.36 | n.s. | TCA cycle |
| Alpha-ketoglutarate dehydrogenase component 4 | P82909 | 0.30 | n.s. | TCA cycle |
| Succinyl-CoA ligase [ADP/GDP-forming] subunit alpha | P53597 | 0.45 | n.s. | TCA cycle |
| Glyoxylate reductase/hydroxypyruvate reductase | Q9UBQ7 | 0.38 | n.s. | Dicarboxylic acid and carboxylic acid metabolism |
Table S1 (cont.)

## Slide 5
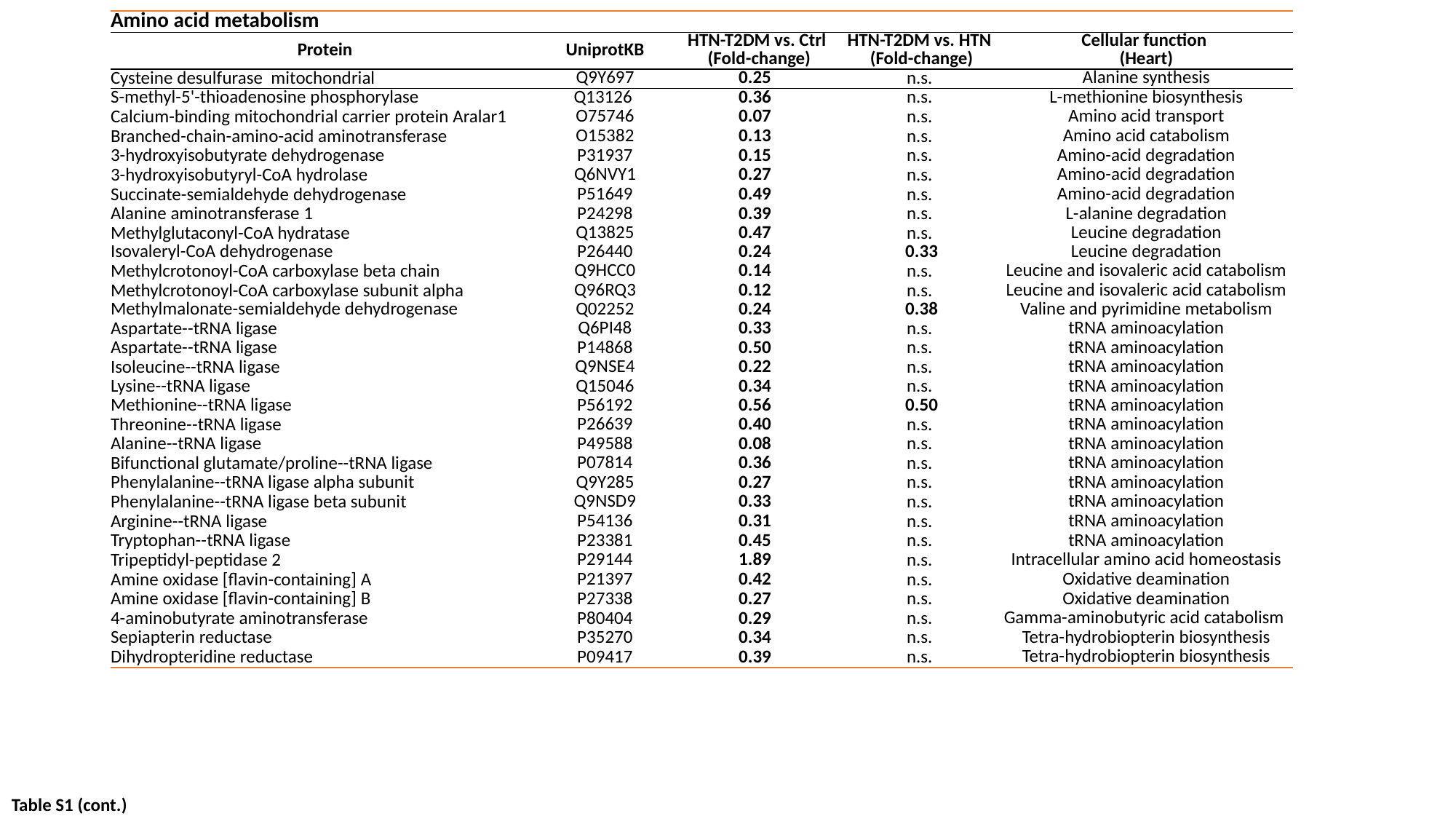

| Amino acid metabolism | | | | |
| --- | --- | --- | --- | --- |
| Protein | UniprotKB | HTN-T2DM vs. Ctrl (Fold-change) | HTN-T2DM vs. HTN (Fold-change) | Cellular function (Heart) |
| Cysteine desulfurase mitochondrial | Q9Y697 | 0.25 | n.s. | Alanine synthesis |
| S-methyl-5'-thioadenosine phosphorylase | Q13126 | 0.36 | n.s. | L-methionine biosynthesis |
| Calcium-binding mitochondrial carrier protein Aralar1 | O75746 | 0.07 | n.s. | Amino acid transport |
| Branched-chain-amino-acid aminotransferase | O15382 | 0.13 | n.s. | Amino acid catabolism |
| 3-hydroxyisobutyrate dehydrogenase | P31937 | 0.15 | n.s. | Amino-acid degradation |
| 3-hydroxyisobutyryl-CoA hydrolase | Q6NVY1 | 0.27 | n.s. | Amino-acid degradation |
| Succinate-semialdehyde dehydrogenase | P51649 | 0.49 | n.s. | Amino-acid degradation |
| Alanine aminotransferase 1 | P24298 | 0.39 | n.s. | L-alanine degradation |
| Methylglutaconyl-CoA hydratase | Q13825 | 0.47 | n.s. | Leucine degradation |
| Isovaleryl-CoA dehydrogenase | P26440 | 0.24 | 0.33 | Leucine degradation |
| Methylcrotonoyl-CoA carboxylase beta chain | Q9HCC0 | 0.14 | n.s. | Leucine and isovaleric acid catabolism |
| Methylcrotonoyl-CoA carboxylase subunit alpha | Q96RQ3 | 0.12 | n.s. | Leucine and isovaleric acid catabolism |
| Methylmalonate-semialdehyde dehydrogenase | Q02252 | 0.24 | 0.38 | Valine and pyrimidine metabolism |
| Aspartate--tRNA ligase | Q6PI48 | 0.33 | n.s. | tRNA aminoacylation |
| Aspartate--tRNA ligase | P14868 | 0.50 | n.s. | tRNA aminoacylation |
| Isoleucine--tRNA ligase | Q9NSE4 | 0.22 | n.s. | tRNA aminoacylation |
| Lysine--tRNA ligase | Q15046 | 0.34 | n.s. | tRNA aminoacylation |
| Methionine--tRNA ligase | P56192 | 0.56 | 0.50 | tRNA aminoacylation |
| Threonine--tRNA ligase | P26639 | 0.40 | n.s. | tRNA aminoacylation |
| Alanine--tRNA ligase | P49588 | 0.08 | n.s. | tRNA aminoacylation |
| Bifunctional glutamate/proline--tRNA ligase | P07814 | 0.36 | n.s. | tRNA aminoacylation |
| Phenylalanine--tRNA ligase alpha subunit | Q9Y285 | 0.27 | n.s. | tRNA aminoacylation |
| Phenylalanine--tRNA ligase beta subunit | Q9NSD9 | 0.33 | n.s. | tRNA aminoacylation |
| Arginine--tRNA ligase | P54136 | 0.31 | n.s. | tRNA aminoacylation |
| Tryptophan--tRNA ligase | P23381 | 0.45 | n.s. | tRNA aminoacylation |
| Tripeptidyl-peptidase 2 | P29144 | 1.89 | n.s. | Intracellular amino acid homeostasis |
| Amine oxidase [flavin-containing] A | P21397 | 0.42 | n.s. | Oxidative deamination |
| Amine oxidase [flavin-containing] B | P27338 | 0.27 | n.s. | Oxidative deamination |
| 4-aminobutyrate aminotransferase | P80404 | 0.29 | n.s. | Gamma-aminobutyric acid catabolism |
| Sepiapterin reductase | P35270 | 0.34 | n.s. | Tetra-hydrobiopterin biosynthesis |
| Dihydropteridine reductase | P09417 | 0.39 | n.s. | Tetra-hydrobiopterin biosynthesis |
Table S1 (cont.)

## Slide 6
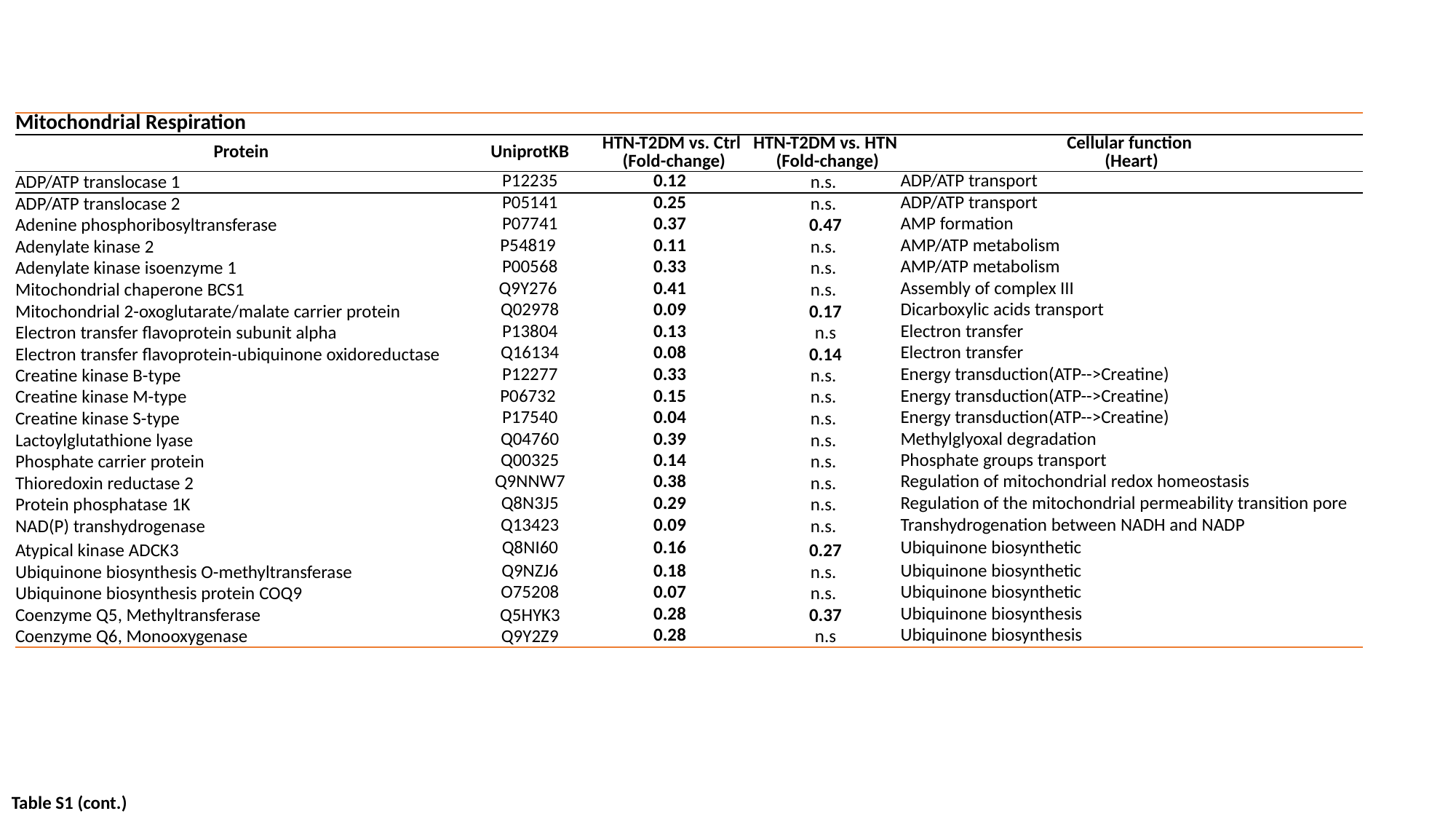

| Mitochondrial Respiration | | | | |
| --- | --- | --- | --- | --- |
| Protein | UniprotKB | HTN-T2DM vs. Ctrl (Fold-change) | HTN-T2DM vs. HTN (Fold-change) | Cellular function (Heart) |
| ADP/ATP translocase 1 | P12235 | 0.12 | n.s. | ADP/ATP transport |
| ADP/ATP translocase 2 | P05141 | 0.25 | n.s. | ADP/ATP transport |
| Adenine phosphoribosyltransferase | P07741 | 0.37 | 0.47 | AMP formation |
| Adenylate kinase 2 | P54819 | 0.11 | n.s. | AMP/ATP metabolism |
| Adenylate kinase isoenzyme 1 | P00568 | 0.33 | n.s. | AMP/ATP metabolism |
| Mitochondrial chaperone BCS1 | Q9Y276 | 0.41 | n.s. | Assembly of complex III |
| Mitochondrial 2-oxoglutarate/malate carrier protein | Q02978 | 0.09 | 0.17 | Dicarboxylic acids transport |
| Electron transfer flavoprotein subunit alpha | P13804 | 0.13 | n.s | Electron transfer |
| Electron transfer flavoprotein-ubiquinone oxidoreductase | Q16134 | 0.08 | 0.14 | Electron transfer |
| Creatine kinase B-type | P12277 | 0.33 | n.s. | Energy transduction(ATP-->Creatine) |
| Creatine kinase M-type | P06732 | 0.15 | n.s. | Energy transduction(ATP-->Creatine) |
| Creatine kinase S-type | P17540 | 0.04 | n.s. | Energy transduction(ATP-->Creatine) |
| Lactoylglutathione lyase | Q04760 | 0.39 | n.s. | Methylglyoxal degradation |
| Phosphate carrier protein | Q00325 | 0.14 | n.s. | Phosphate groups transport |
| Thioredoxin reductase 2 | Q9NNW7 | 0.38 | n.s. | Regulation of mitochondrial redox homeostasis |
| Protein phosphatase 1K | Q8N3J5 | 0.29 | n.s. | Regulation of the mitochondrial permeability transition pore |
| NAD(P) transhydrogenase | Q13423 | 0.09 | n.s. | Transhydrogenation between NADH and NADP |
| Atypical kinase ADCK3 | Q8NI60 | 0.16 | 0.27 | Ubiquinone biosynthetic |
| Ubiquinone biosynthesis O-methyltransferase | Q9NZJ6 | 0.18 | n.s. | Ubiquinone biosynthetic |
| Ubiquinone biosynthesis protein COQ9 | O75208 | 0.07 | n.s. | Ubiquinone biosynthetic |
| Coenzyme Q5, Methyltransferase | Q5HYK3 | 0.28 | 0.37 | Ubiquinone biosynthesis |
| Coenzyme Q6, Monooxygenase | Q9Y2Z9 | 0.28 | n.s | Ubiquinone biosynthesis |
Table S1 (cont.)

## Slide 7
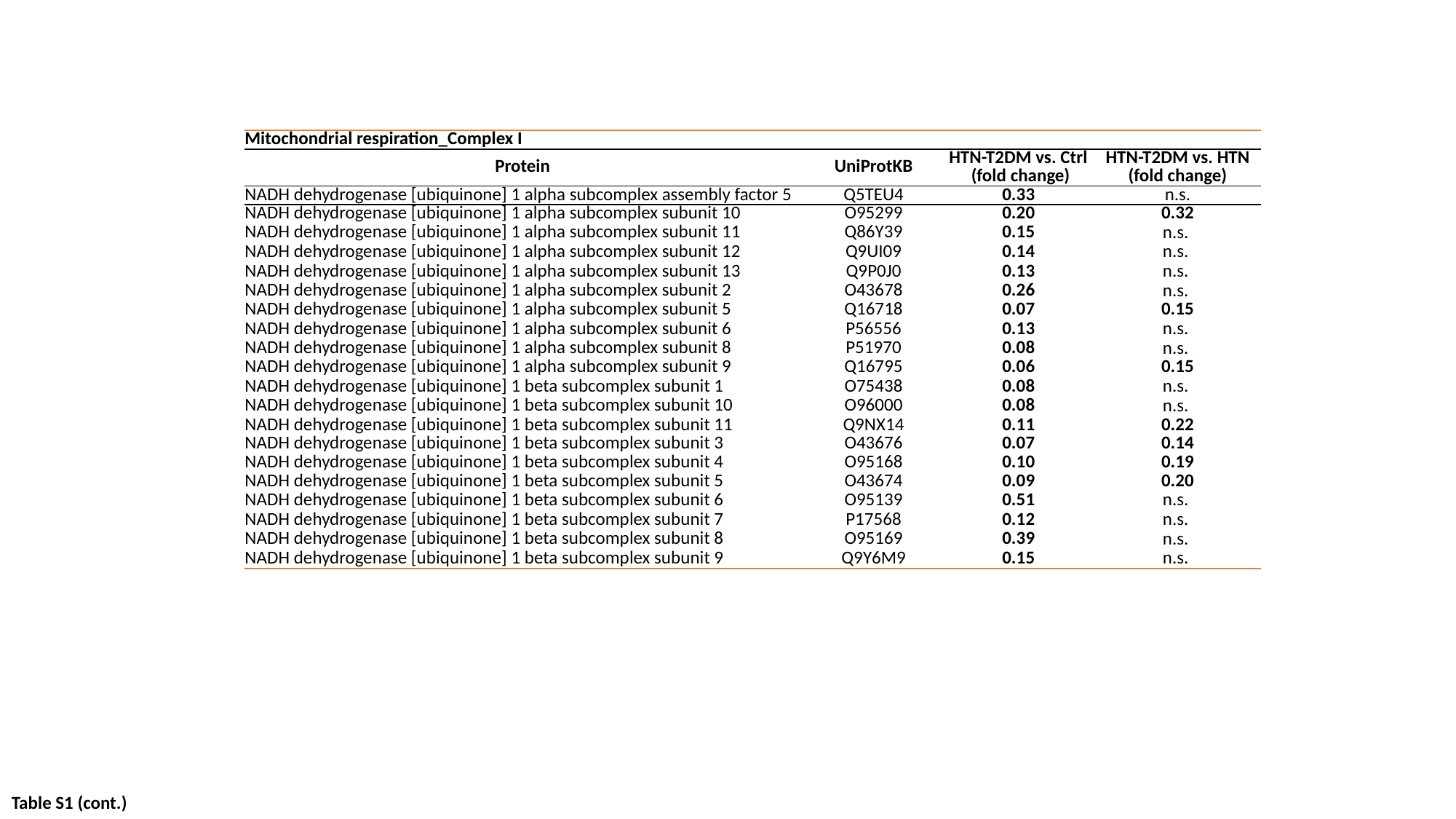

| Mitochondrial respiration\_Complex I | | | |
| --- | --- | --- | --- |
| Protein | UniProtKB | HTN-T2DM vs. Ctrl (fold change) | HTN-T2DM vs. HTN (fold change) |
| NADH dehydrogenase [ubiquinone] 1 alpha subcomplex assembly factor 5 | Q5TEU4 | 0.33 | n.s. |
| NADH dehydrogenase [ubiquinone] 1 alpha subcomplex subunit 10 | O95299 | 0.20 | 0.32 |
| NADH dehydrogenase [ubiquinone] 1 alpha subcomplex subunit 11 | Q86Y39 | 0.15 | n.s. |
| NADH dehydrogenase [ubiquinone] 1 alpha subcomplex subunit 12 | Q9UI09 | 0.14 | n.s. |
| NADH dehydrogenase [ubiquinone] 1 alpha subcomplex subunit 13 | Q9P0J0 | 0.13 | n.s. |
| NADH dehydrogenase [ubiquinone] 1 alpha subcomplex subunit 2 | O43678 | 0.26 | n.s. |
| NADH dehydrogenase [ubiquinone] 1 alpha subcomplex subunit 5 | Q16718 | 0.07 | 0.15 |
| NADH dehydrogenase [ubiquinone] 1 alpha subcomplex subunit 6 | P56556 | 0.13 | n.s. |
| NADH dehydrogenase [ubiquinone] 1 alpha subcomplex subunit 8 | P51970 | 0.08 | n.s. |
| NADH dehydrogenase [ubiquinone] 1 alpha subcomplex subunit 9 | Q16795 | 0.06 | 0.15 |
| NADH dehydrogenase [ubiquinone] 1 beta subcomplex subunit 1 | O75438 | 0.08 | n.s. |
| NADH dehydrogenase [ubiquinone] 1 beta subcomplex subunit 10 | O96000 | 0.08 | n.s. |
| NADH dehydrogenase [ubiquinone] 1 beta subcomplex subunit 11 | Q9NX14 | 0.11 | 0.22 |
| NADH dehydrogenase [ubiquinone] 1 beta subcomplex subunit 3 | O43676 | 0.07 | 0.14 |
| NADH dehydrogenase [ubiquinone] 1 beta subcomplex subunit 4 | O95168 | 0.10 | 0.19 |
| NADH dehydrogenase [ubiquinone] 1 beta subcomplex subunit 5 | O43674 | 0.09 | 0.20 |
| NADH dehydrogenase [ubiquinone] 1 beta subcomplex subunit 6 | O95139 | 0.51 | n.s. |
| NADH dehydrogenase [ubiquinone] 1 beta subcomplex subunit 7 | P17568 | 0.12 | n.s. |
| NADH dehydrogenase [ubiquinone] 1 beta subcomplex subunit 8 | O95169 | 0.39 | n.s. |
| NADH dehydrogenase [ubiquinone] 1 beta subcomplex subunit 9 | Q9Y6M9 | 0.15 | n.s. |
Table S1 (cont.)

## Slide 8
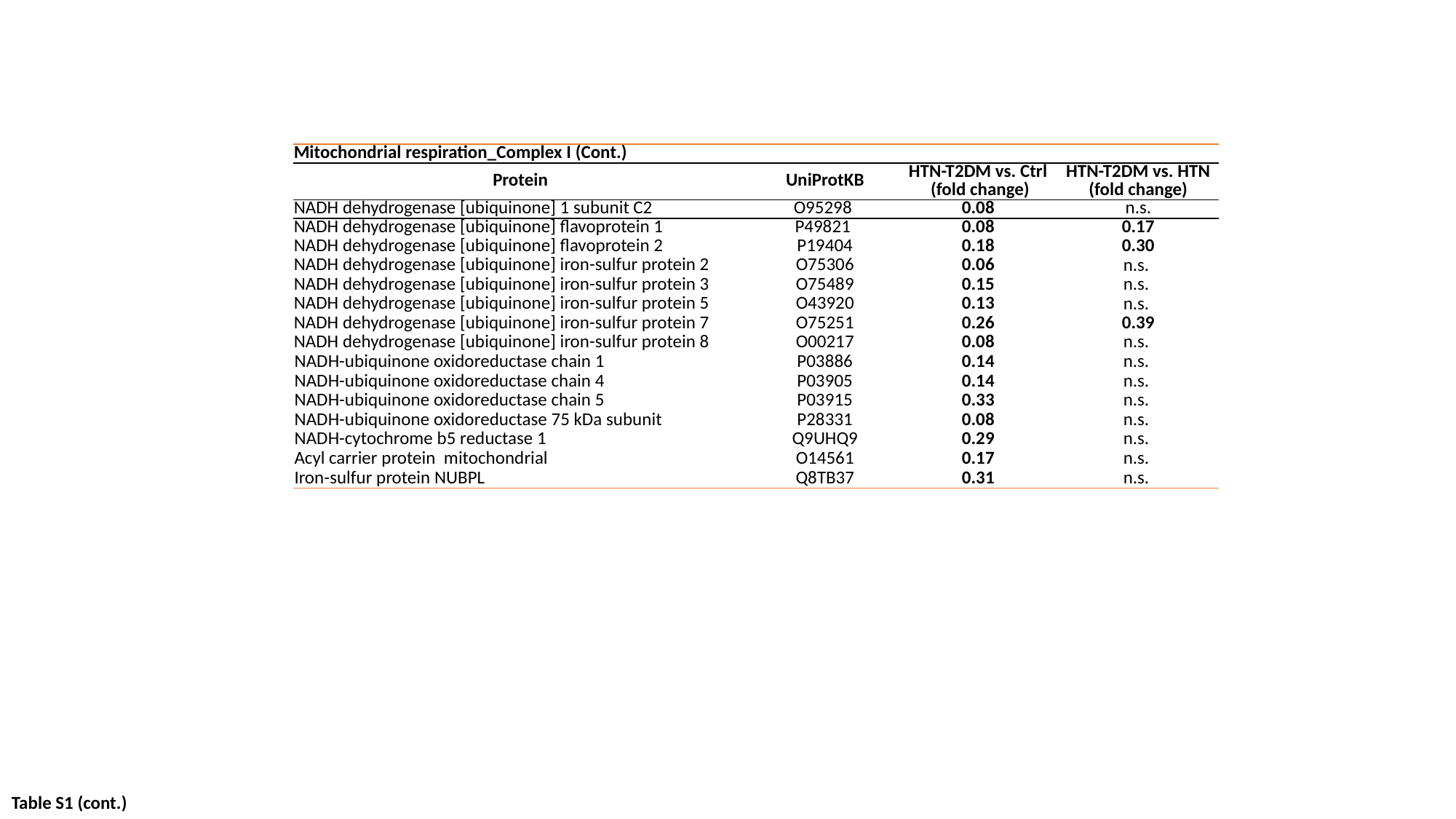

| Mitochondrial respiration\_Complex I (Cont.) | | | |
| --- | --- | --- | --- |
| Protein | UniProtKB | HTN-T2DM vs. Ctrl (fold change) | HTN-T2DM vs. HTN (fold change) |
| NADH dehydrogenase [ubiquinone] 1 subunit C2 | O95298 | 0.08 | n.s. |
| NADH dehydrogenase [ubiquinone] flavoprotein 1 | P49821 | 0.08 | 0.17 |
| NADH dehydrogenase [ubiquinone] flavoprotein 2 | P19404 | 0.18 | 0.30 |
| NADH dehydrogenase [ubiquinone] iron-sulfur protein 2 | O75306 | 0.06 | n.s. |
| NADH dehydrogenase [ubiquinone] iron-sulfur protein 3 | O75489 | 0.15 | n.s. |
| NADH dehydrogenase [ubiquinone] iron-sulfur protein 5 | O43920 | 0.13 | n.s. |
| NADH dehydrogenase [ubiquinone] iron-sulfur protein 7 | O75251 | 0.26 | 0.39 |
| NADH dehydrogenase [ubiquinone] iron-sulfur protein 8 | O00217 | 0.08 | n.s. |
| NADH-ubiquinone oxidoreductase chain 1 | P03886 | 0.14 | n.s. |
| NADH-ubiquinone oxidoreductase chain 4 | P03905 | 0.14 | n.s. |
| NADH-ubiquinone oxidoreductase chain 5 | P03915 | 0.33 | n.s. |
| NADH-ubiquinone oxidoreductase 75 kDa subunit | P28331 | 0.08 | n.s. |
| NADH-cytochrome b5 reductase 1 | Q9UHQ9 | 0.29 | n.s. |
| Acyl carrier protein mitochondrial | O14561 | 0.17 | n.s. |
| Iron-sulfur protein NUBPL | Q8TB37 | 0.31 | n.s. |
Table S1 (cont.)

## Slide 9
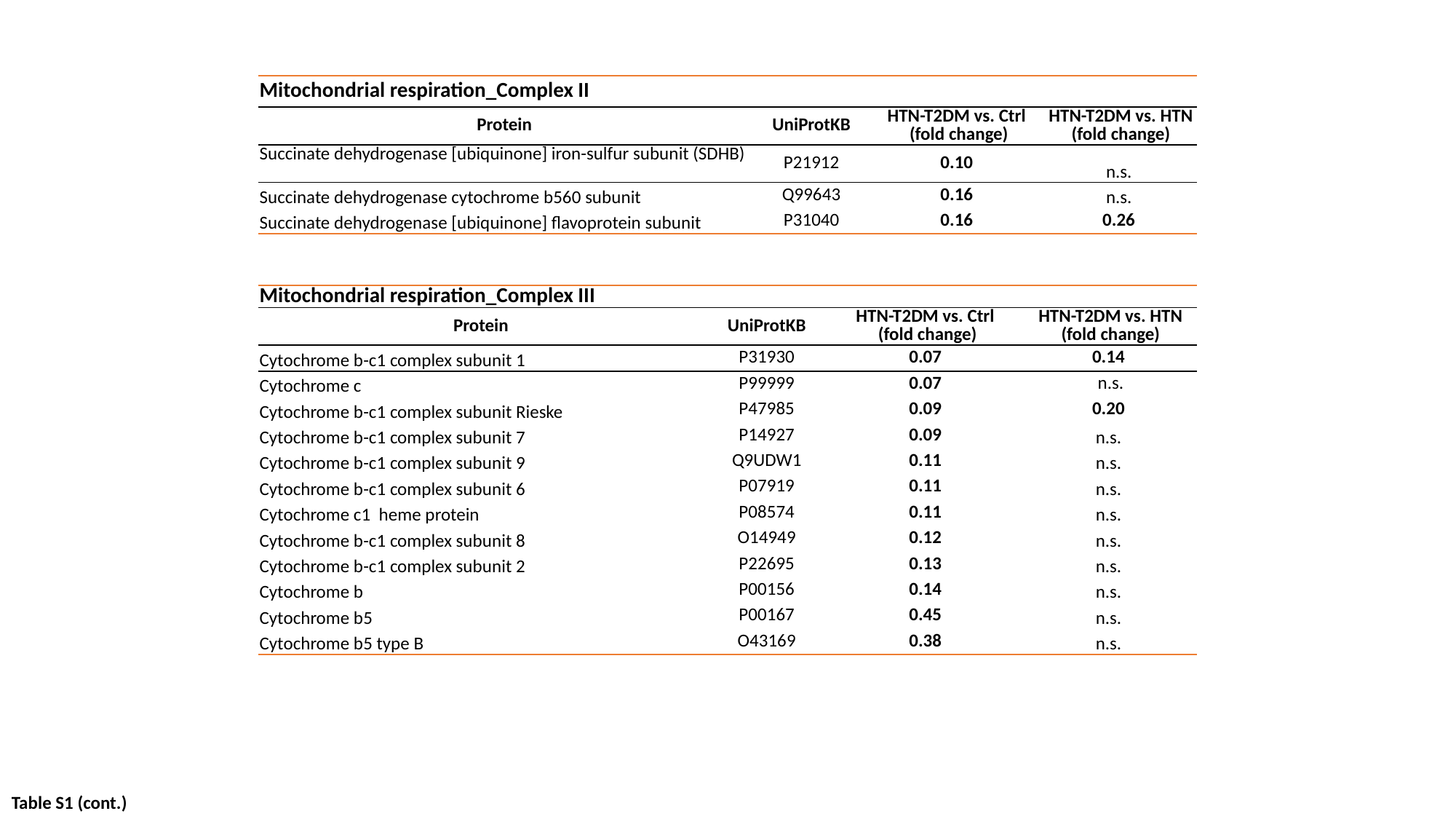

| Mitochondrial respiration\_Complex II | | | |
| --- | --- | --- | --- |
| Protein | UniProtKB | HTN-T2DM vs. Ctrl (fold change) | HTN-T2DM vs. HTN (fold change) |
| Succinate dehydrogenase [ubiquinone] iron-sulfur subunit (SDHB) | P21912 | 0.10 | n.s. |
| Succinate dehydrogenase cytochrome b560 subunit | Q99643 | 0.16 | n.s. |
| Succinate dehydrogenase [ubiquinone] flavoprotein subunit | P31040 | 0.16 | 0.26 |
| Mitochondrial respiration\_Complex III | | | |
| --- | --- | --- | --- |
| Protein | UniProtKB | HTN-T2DM vs. Ctrl (fold change) | HTN-T2DM vs. HTN (fold change) |
| Cytochrome b-c1 complex subunit 1 | P31930 | 0.07 | 0.14 |
| Cytochrome c | P99999 | 0.07 | n.s. |
| Cytochrome b-c1 complex subunit Rieske | P47985 | 0.09 | 0.20 |
| Cytochrome b-c1 complex subunit 7 | P14927 | 0.09 | n.s. |
| Cytochrome b-c1 complex subunit 9 | Q9UDW1 | 0.11 | n.s. |
| Cytochrome b-c1 complex subunit 6 | P07919 | 0.11 | n.s. |
| Cytochrome c1 heme protein | P08574 | 0.11 | n.s. |
| Cytochrome b-c1 complex subunit 8 | O14949 | 0.12 | n.s. |
| Cytochrome b-c1 complex subunit 2 | P22695 | 0.13 | n.s. |
| Cytochrome b | P00156 | 0.14 | n.s. |
| Cytochrome b5 | P00167 | 0.45 | n.s. |
| Cytochrome b5 type B | O43169 | 0.38 | n.s. |
Table S1 (cont.)

## Slide 10
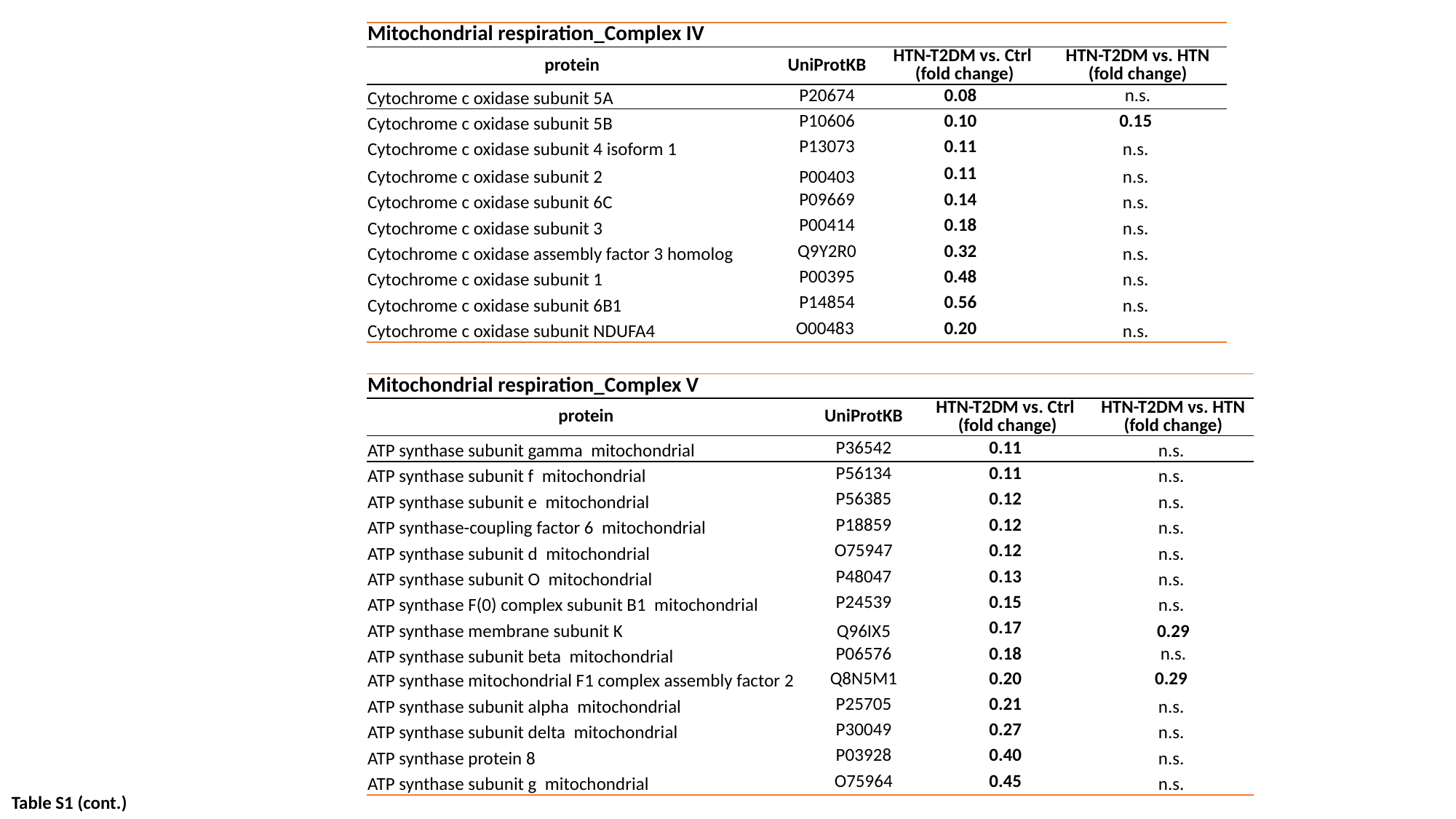

| Mitochondrial respiration\_Complex IV | | | |
| --- | --- | --- | --- |
| protein | UniProtKB | HTN-T2DM vs. Ctrl (fold change) | HTN-T2DM vs. HTN (fold change) |
| Cytochrome c oxidase subunit 5A | P20674 | 0.08 | n.s. |
| Cytochrome c oxidase subunit 5B | P10606 | 0.10 | 0.15 |
| Cytochrome c oxidase subunit 4 isoform 1 | P13073 | 0.11 | n.s. |
| Cytochrome c oxidase subunit 2 | P00403 | 0.11 | n.s. |
| Cytochrome c oxidase subunit 6C | P09669 | 0.14 | n.s. |
| Cytochrome c oxidase subunit 3 | P00414 | 0.18 | n.s. |
| Cytochrome c oxidase assembly factor 3 homolog | Q9Y2R0 | 0.32 | n.s. |
| Cytochrome c oxidase subunit 1 | P00395 | 0.48 | n.s. |
| Cytochrome c oxidase subunit 6B1 | P14854 | 0.56 | n.s. |
| Cytochrome c oxidase subunit NDUFA4 | O00483 | 0.20 | n.s. |
| Mitochondrial respiration\_Complex V | | | |
| --- | --- | --- | --- |
| protein | UniProtKB | HTN-T2DM vs. Ctrl (fold change) | HTN-T2DM vs. HTN (fold change) |
| ATP synthase subunit gamma mitochondrial | P36542 | 0.11 | n.s. |
| ATP synthase subunit f mitochondrial | P56134 | 0.11 | n.s. |
| ATP synthase subunit e mitochondrial | P56385 | 0.12 | n.s. |
| ATP synthase-coupling factor 6 mitochondrial | P18859 | 0.12 | n.s. |
| ATP synthase subunit d mitochondrial | O75947 | 0.12 | n.s. |
| ATP synthase subunit O mitochondrial | P48047 | 0.13 | n.s. |
| ATP synthase F(0) complex subunit B1 mitochondrial | P24539 | 0.15 | n.s. |
| ATP synthase membrane subunit K | Q96IX5 | 0.17 | 0.29 |
| ATP synthase subunit beta mitochondrial | P06576 | 0.18 | n.s. |
| ATP synthase mitochondrial F1 complex assembly factor 2 | Q8N5M1 | 0.20 | 0.29 |
| ATP synthase subunit alpha mitochondrial | P25705 | 0.21 | n.s. |
| ATP synthase subunit delta mitochondrial | P30049 | 0.27 | n.s. |
| ATP synthase protein 8 | P03928 | 0.40 | n.s. |
| ATP synthase subunit g mitochondrial | O75964 | 0.45 | n.s. |
Table S1 (cont.)

## Slide 11
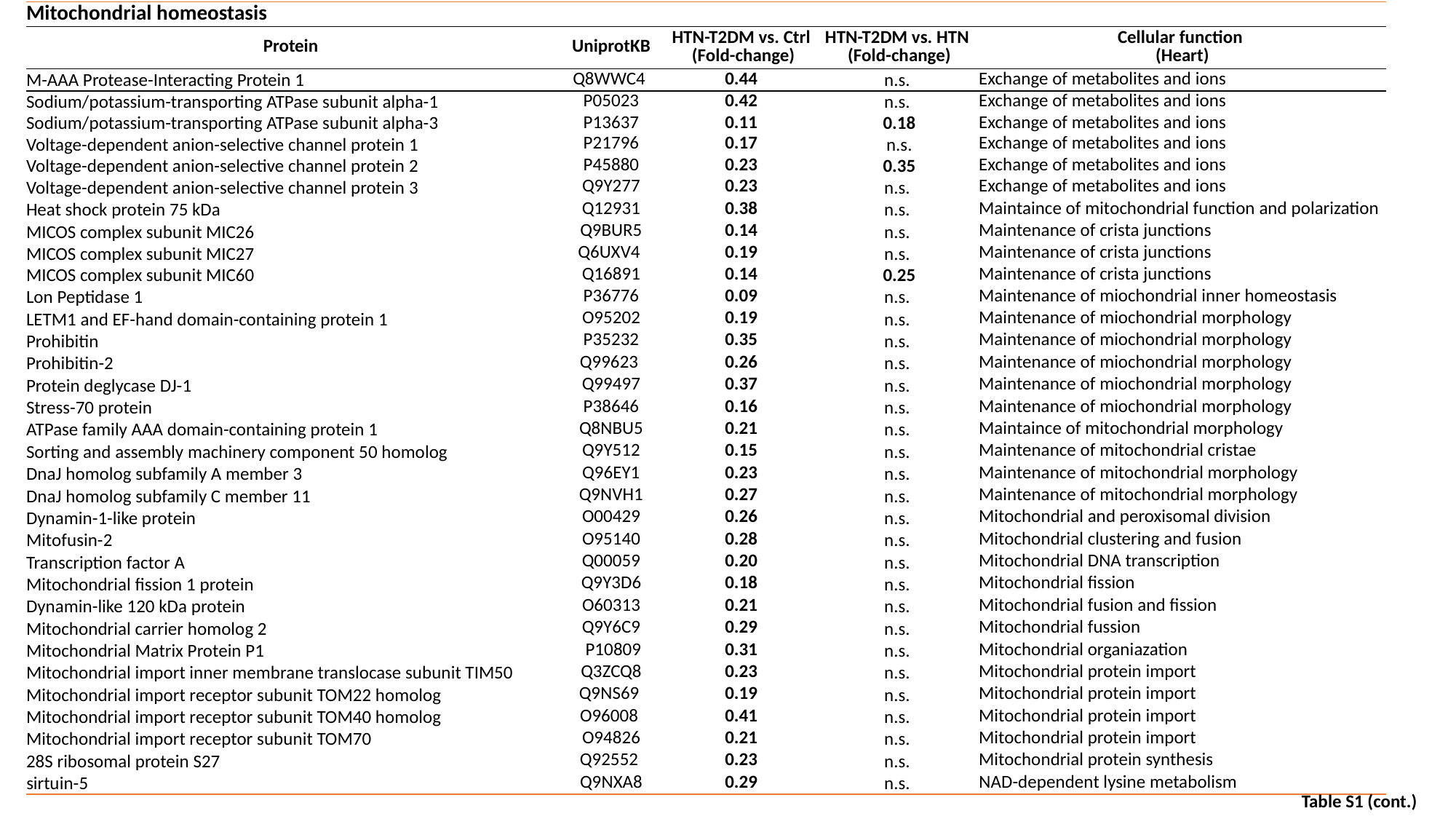

| Mitochondrial homeostasis | | | | |
| --- | --- | --- | --- | --- |
| Protein | UniprotKB | HTN-T2DM vs. Ctrl (Fold-change) | HTN-T2DM vs. HTN (Fold-change) | Cellular function (Heart) |
| M-AAA Protease-Interacting Protein 1 | Q8WWC4 | 0.44 | n.s. | Exchange of metabolites and ions |
| Sodium/potassium-transporting ATPase subunit alpha-1 | P05023 | 0.42 | n.s. | Exchange of metabolites and ions |
| Sodium/potassium-transporting ATPase subunit alpha-3 | P13637 | 0.11 | 0.18 | Exchange of metabolites and ions |
| Voltage-dependent anion-selective channel protein 1 | P21796 | 0.17 | n.s. | Exchange of metabolites and ions |
| Voltage-dependent anion-selective channel protein 2 | P45880 | 0.23 | 0.35 | Exchange of metabolites and ions |
| Voltage-dependent anion-selective channel protein 3 | Q9Y277 | 0.23 | n.s. | Exchange of metabolites and ions |
| Heat shock protein 75 kDa | Q12931 | 0.38 | n.s. | Maintaince of mitochondrial function and polarization |
| MICOS complex subunit MIC26 | Q9BUR5 | 0.14 | n.s. | Maintenance of crista junctions |
| MICOS complex subunit MIC27 | Q6UXV4 | 0.19 | n.s. | Maintenance of crista junctions |
| MICOS complex subunit MIC60 | Q16891 | 0.14 | 0.25 | Maintenance of crista junctions |
| Lon Peptidase 1 | P36776 | 0.09 | n.s. | Maintenance of miochondrial inner homeostasis |
| LETM1 and EF-hand domain-containing protein 1 | O95202 | 0.19 | n.s. | Maintenance of miochondrial morphology |
| Prohibitin | P35232 | 0.35 | n.s. | Maintenance of miochondrial morphology |
| Prohibitin-2 | Q99623 | 0.26 | n.s. | Maintenance of miochondrial morphology |
| Protein deglycase DJ-1 | Q99497 | 0.37 | n.s. | Maintenance of miochondrial morphology |
| Stress-70 protein | P38646 | 0.16 | n.s. | Maintenance of miochondrial morphology |
| ATPase family AAA domain-containing protein 1 | Q8NBU5 | 0.21 | n.s. | Maintaince of mitochondrial morphology |
| Sorting and assembly machinery component 50 homolog | Q9Y512 | 0.15 | n.s. | Maintenance of mitochondrial cristae |
| DnaJ homolog subfamily A member 3 | Q96EY1 | 0.23 | n.s. | Maintenance of mitochondrial morphology |
| DnaJ homolog subfamily C member 11 | Q9NVH1 | 0.27 | n.s. | Maintenance of mitochondrial morphology |
| Dynamin-1-like protein | O00429 | 0.26 | n.s. | Mitochondrial and peroxisomal division |
| Mitofusin-2 | O95140 | 0.28 | n.s. | Mitochondrial clustering and fusion |
| Transcription factor A | Q00059 | 0.20 | n.s. | Mitochondrial DNA transcription |
| Mitochondrial fission 1 protein | Q9Y3D6 | 0.18 | n.s. | Mitochondrial fission |
| Dynamin-like 120 kDa protein | O60313 | 0.21 | n.s. | Mitochondrial fusion and fission |
| Mitochondrial carrier homolog 2 | Q9Y6C9 | 0.29 | n.s. | Mitochondrial fussion |
| Mitochondrial Matrix Protein P1 | P10809 | 0.31 | n.s. | Mitochondrial organiazation |
| Mitochondrial import inner membrane translocase subunit TIM50 | Q3ZCQ8 | 0.23 | n.s. | Mitochondrial protein import |
| Mitochondrial import receptor subunit TOM22 homolog | Q9NS69 | 0.19 | n.s. | Mitochondrial protein import |
| Mitochondrial import receptor subunit TOM40 homolog | O96008 | 0.41 | n.s. | Mitochondrial protein import |
| Mitochondrial import receptor subunit TOM70 | O94826 | 0.21 | n.s. | Mitochondrial protein import |
| 28S ribosomal protein S27 | Q92552 | 0.23 | n.s. | Mitochondrial protein synthesis |
| sirtuin-5 | Q9NXA8 | 0.29 | n.s. | NAD-dependent lysine metabolism |
Table S1 (cont.)

## Slide 12
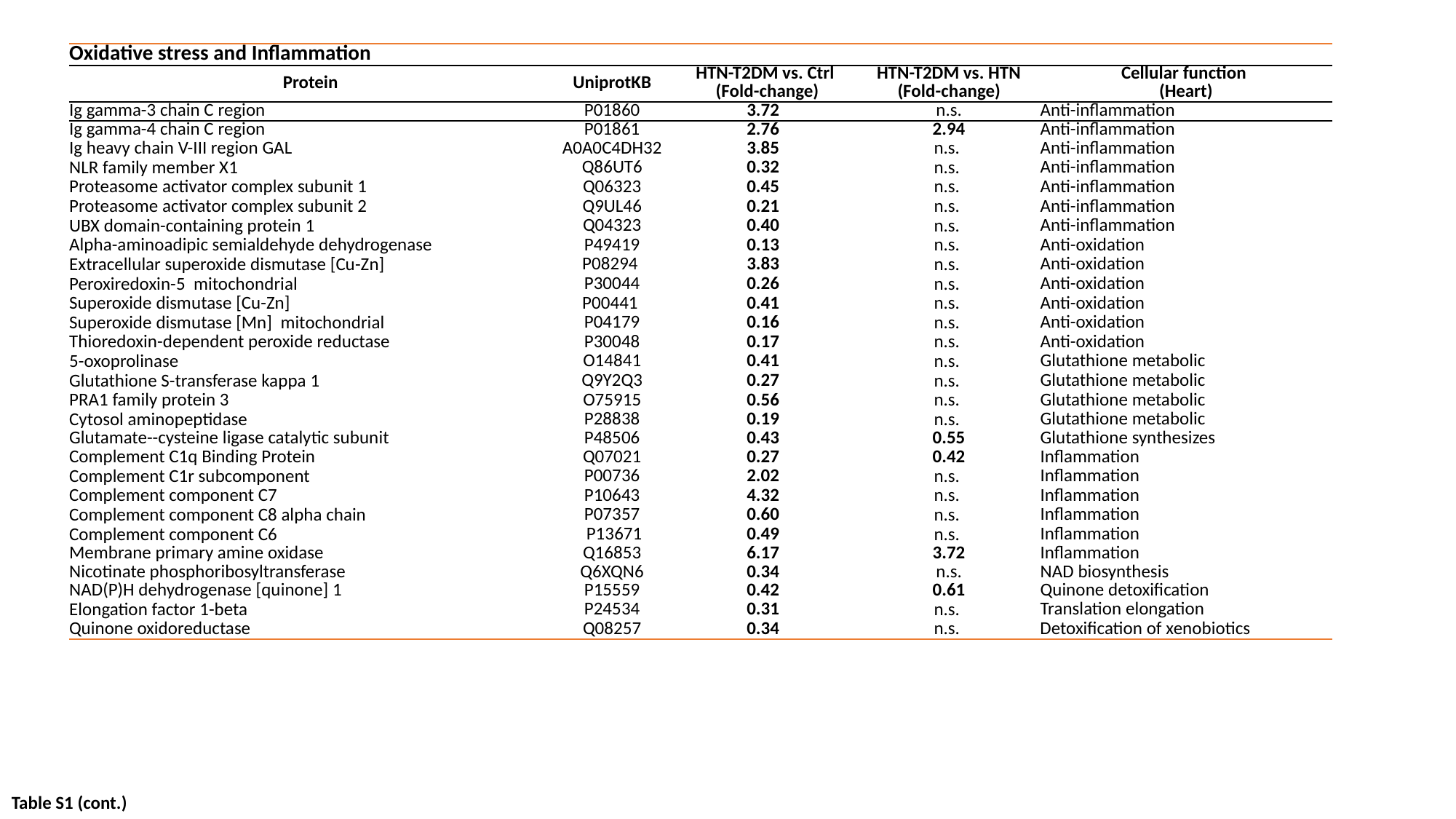

| Oxidative stress and Inflammation | | | | |
| --- | --- | --- | --- | --- |
| Protein | UniprotKB | HTN-T2DM vs. Ctrl (Fold-change) | HTN-T2DM vs. HTN (Fold-change) | Cellular function (Heart) |
| Ig gamma-3 chain C region | P01860 | 3.72 | n.s. | Anti-inflammation |
| Ig gamma-4 chain C region | P01861 | 2.76 | 2.94 | Anti-inflammation |
| Ig heavy chain V-III region GAL | A0A0C4DH32 | 3.85 | n.s. | Anti-inflammation |
| NLR family member X1 | Q86UT6 | 0.32 | n.s. | Anti-inflammation |
| Proteasome activator complex subunit 1 | Q06323 | 0.45 | n.s. | Anti-inflammation |
| Proteasome activator complex subunit 2 | Q9UL46 | 0.21 | n.s. | Anti-inflammation |
| UBX domain-containing protein 1 | Q04323 | 0.40 | n.s. | Anti-inflammation |
| Alpha-aminoadipic semialdehyde dehydrogenase | P49419 | 0.13 | n.s. | Anti-oxidation |
| Extracellular superoxide dismutase [Cu-Zn] | P08294 | 3.83 | n.s. | Anti-oxidation |
| Peroxiredoxin-5 mitochondrial | P30044 | 0.26 | n.s. | Anti-oxidation |
| Superoxide dismutase [Cu-Zn] | P00441 | 0.41 | n.s. | Anti-oxidation |
| Superoxide dismutase [Mn] mitochondrial | P04179 | 0.16 | n.s. | Anti-oxidation |
| Thioredoxin-dependent peroxide reductase | P30048 | 0.17 | n.s. | Anti-oxidation |
| 5-oxoprolinase | O14841 | 0.41 | n.s. | Glutathione metabolic |
| Glutathione S-transferase kappa 1 | Q9Y2Q3 | 0.27 | n.s. | Glutathione metabolic |
| PRA1 family protein 3 | O75915 | 0.56 | n.s. | Glutathione metabolic |
| Cytosol aminopeptidase | P28838 | 0.19 | n.s. | Glutathione metabolic |
| Glutamate--cysteine ligase catalytic subunit | P48506 | 0.43 | 0.55 | Glutathione synthesizes |
| Complement C1q Binding Protein | Q07021 | 0.27 | 0.42 | Inflammation |
| Complement C1r subcomponent | P00736 | 2.02 | n.s. | Inflammation |
| Complement component C7 | P10643 | 4.32 | n.s. | Inflammation |
| Complement component C8 alpha chain | P07357 | 0.60 | n.s. | Inflammation |
| Complement component C6 | P13671 | 0.49 | n.s. | Inflammation |
| Membrane primary amine oxidase | Q16853 | 6.17 | 3.72 | Inflammation |
| Nicotinate phosphoribosyltransferase | Q6XQN6 | 0.34 | n.s. | NAD biosynthesis |
| NAD(P)H dehydrogenase [quinone] 1 | P15559 | 0.42 | 0.61 | Quinone detoxification |
| Elongation factor 1-beta | P24534 | 0.31 | n.s. | Translation elongation |
| Quinone oxidoreductase | Q08257 | 0.34 | n.s. | Detoxification of xenobiotics |
Table S1 (cont.)

## Slide 13
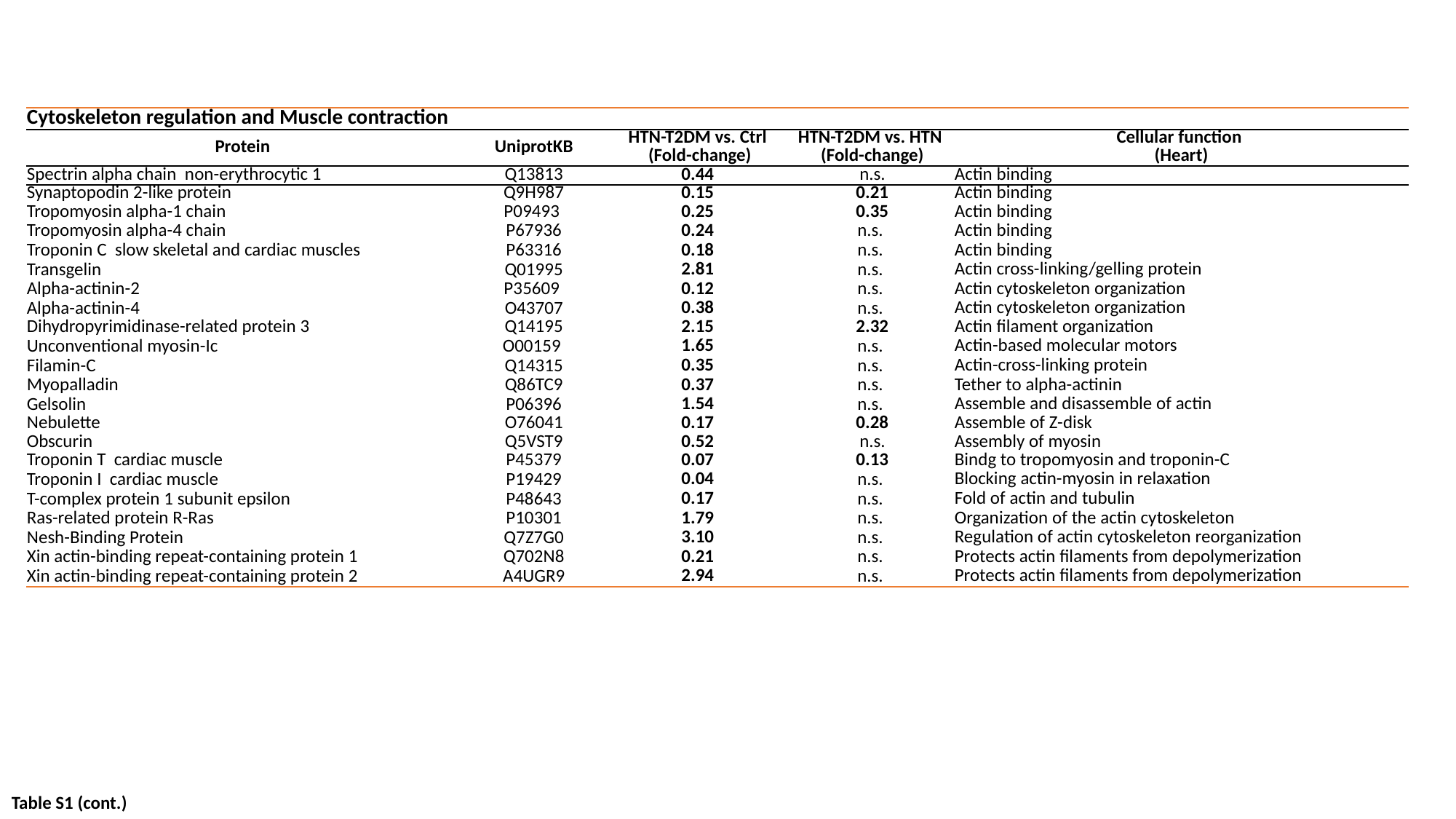

| Cytoskeleton regulation and Muscle contraction | | | | |
| --- | --- | --- | --- | --- |
| Protein | UniprotKB | HTN-T2DM vs. Ctrl (Fold-change) | HTN-T2DM vs. HTN (Fold-change) | Cellular function (Heart) |
| Spectrin alpha chain non-erythrocytic 1 | Q13813 | 0.44 | n.s. | Actin binding |
| Synaptopodin 2-like protein | Q9H987 | 0.15 | 0.21 | Actin binding |
| Tropomyosin alpha-1 chain | P09493 | 0.25 | 0.35 | Actin binding |
| Tropomyosin alpha-4 chain | P67936 | 0.24 | n.s. | Actin binding |
| Troponin C slow skeletal and cardiac muscles | P63316 | 0.18 | n.s. | Actin binding |
| Transgelin | Q01995 | 2.81 | n.s. | Actin cross-linking/gelling protein |
| Alpha-actinin-2 | P35609 | 0.12 | n.s. | Actin cytoskeleton organization |
| Alpha-actinin-4 | O43707 | 0.38 | n.s. | Actin cytoskeleton organization |
| Dihydropyrimidinase-related protein 3 | Q14195 | 2.15 | 2.32 | Actin filament organization |
| Unconventional myosin-Ic | O00159 | 1.65 | n.s. | Actin-based molecular motors |
| Filamin-C | Q14315 | 0.35 | n.s. | Actin-cross-linking protein |
| Myopalladin | Q86TC9 | 0.37 | n.s. | Tether to alpha-actinin |
| Gelsolin | P06396 | 1.54 | n.s. | Assemble and disassemble of actin |
| Nebulette | O76041 | 0.17 | 0.28 | Assemble of Z-disk |
| Obscurin | Q5VST9 | 0.52 | n.s. | Assembly of myosin |
| Troponin T cardiac muscle | P45379 | 0.07 | 0.13 | Bindg to tropomyosin and troponin-C |
| Troponin I cardiac muscle | P19429 | 0.04 | n.s. | Blocking actin-myosin in relaxation |
| T-complex protein 1 subunit epsilon | P48643 | 0.17 | n.s. | Fold of actin and tubulin |
| Ras-related protein R-Ras | P10301 | 1.79 | n.s. | Organization of the actin cytoskeleton |
| Nesh-Binding Protein | Q7Z7G0 | 3.10 | n.s. | Regulation of actin cytoskeleton reorganization |
| Xin actin-binding repeat-containing protein 1 | Q702N8 | 0.21 | n.s. | Protects actin filaments from depolymerization |
| Xin actin-binding repeat-containing protein 2 | A4UGR9 | 2.94 | n.s. | Protects actin filaments from depolymerization |
Table S1 (cont.)

## Slide 14
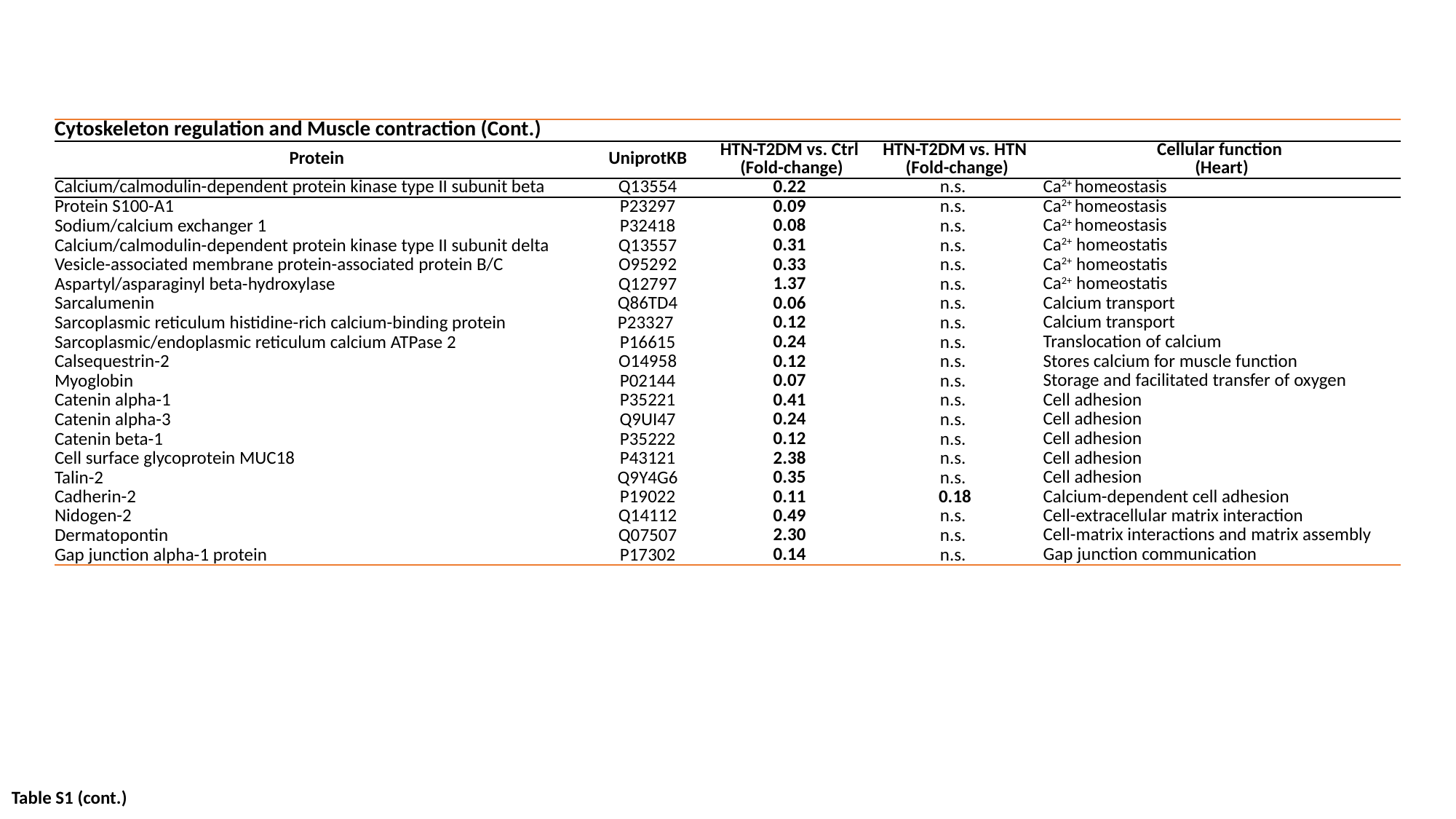

| Cytoskeleton regulation and Muscle contraction (Cont.) | | | | |
| --- | --- | --- | --- | --- |
| Protein | UniprotKB | HTN-T2DM vs. Ctrl (Fold-change) | HTN-T2DM vs. HTN (Fold-change) | Cellular function (Heart) |
| Calcium/calmodulin-dependent protein kinase type II subunit beta | Q13554 | 0.22 | n.s. | Ca2+ homeostasis |
| Protein S100-A1 | P23297 | 0.09 | n.s. | Ca2+ homeostasis |
| Sodium/calcium exchanger 1 | P32418 | 0.08 | n.s. | Ca2+ homeostasis |
| Calcium/calmodulin-dependent protein kinase type II subunit delta | Q13557 | 0.31 | n.s. | Ca2+ homeostatis |
| Vesicle-associated membrane protein-associated protein B/C | O95292 | 0.33 | n.s. | Ca2+ homeostatis |
| Aspartyl/asparaginyl beta-hydroxylase | Q12797 | 1.37 | n.s. | Ca2+ homeostatis |
| Sarcalumenin | Q86TD4 | 0.06 | n.s. | Calcium transport |
| Sarcoplasmic reticulum histidine-rich calcium-binding protein | P23327 | 0.12 | n.s. | Calcium transport |
| Sarcoplasmic/endoplasmic reticulum calcium ATPase 2 | P16615 | 0.24 | n.s. | Translocation of calcium |
| Calsequestrin-2 | O14958 | 0.12 | n.s. | Stores calcium for muscle function |
| Myoglobin | P02144 | 0.07 | n.s. | Storage and facilitated transfer of oxygen |
| Catenin alpha-1 | P35221 | 0.41 | n.s. | Cell adhesion |
| Catenin alpha-3 | Q9UI47 | 0.24 | n.s. | Cell adhesion |
| Catenin beta-1 | P35222 | 0.12 | n.s. | Cell adhesion |
| Cell surface glycoprotein MUC18 | P43121 | 2.38 | n.s. | Cell adhesion |
| Talin-2 | Q9Y4G6 | 0.35 | n.s. | Cell adhesion |
| Cadherin-2 | P19022 | 0.11 | 0.18 | Calcium-dependent cell adhesion |
| Nidogen-2 | Q14112 | 0.49 | n.s. | Cell-extracellular matrix interaction |
| Dermatopontin | Q07507 | 2.30 | n.s. | Cell-matrix interactions and matrix assembly |
| Gap junction alpha-1 protein | P17302 | 0.14 | n.s. | Gap junction communication |
Table S1 (cont.)

## Slide 15
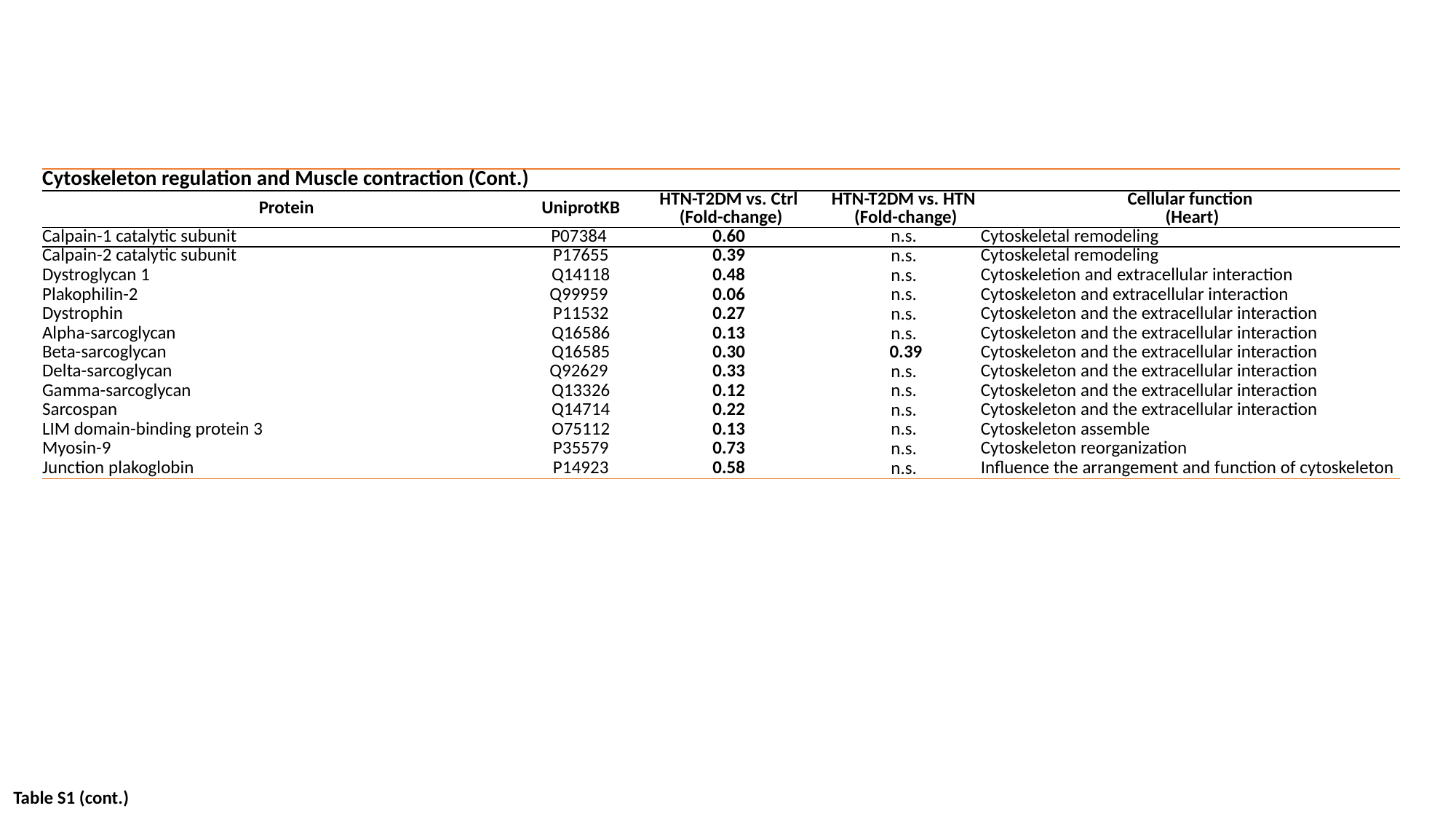

| Cytoskeleton regulation and Muscle contraction (Cont.) | | | | |
| --- | --- | --- | --- | --- |
| Protein | UniprotKB | HTN-T2DM vs. Ctrl (Fold-change) | HTN-T2DM vs. HTN (Fold-change) | Cellular function (Heart) |
| Calpain-1 catalytic subunit | P07384 | 0.60 | n.s. | Cytoskeletal remodeling |
| Calpain-2 catalytic subunit | P17655 | 0.39 | n.s. | Cytoskeletal remodeling |
| Dystroglycan 1 | Q14118 | 0.48 | n.s. | Cytoskeletion and extracellular interaction |
| Plakophilin-2 | Q99959 | 0.06 | n.s. | Cytoskeleton and extracellular interaction |
| Dystrophin | P11532 | 0.27 | n.s. | Cytoskeleton and the extracellular interaction |
| Alpha-sarcoglycan | Q16586 | 0.13 | n.s. | Cytoskeleton and the extracellular interaction |
| Beta-sarcoglycan | Q16585 | 0.30 | 0.39 | Cytoskeleton and the extracellular interaction |
| Delta-sarcoglycan | Q92629 | 0.33 | n.s. | Cytoskeleton and the extracellular interaction |
| Gamma-sarcoglycan | Q13326 | 0.12 | n.s. | Cytoskeleton and the extracellular interaction |
| Sarcospan | Q14714 | 0.22 | n.s. | Cytoskeleton and the extracellular interaction |
| LIM domain-binding protein 3 | O75112 | 0.13 | n.s. | Cytoskeleton assemble |
| Myosin-9 | P35579 | 0.73 | n.s. | Cytoskeleton reorganization |
| Junction plakoglobin | P14923 | 0.58 | n.s. | Influence the arrangement and function of cytoskeleton |
Table S1 (cont.)

## Slide 16
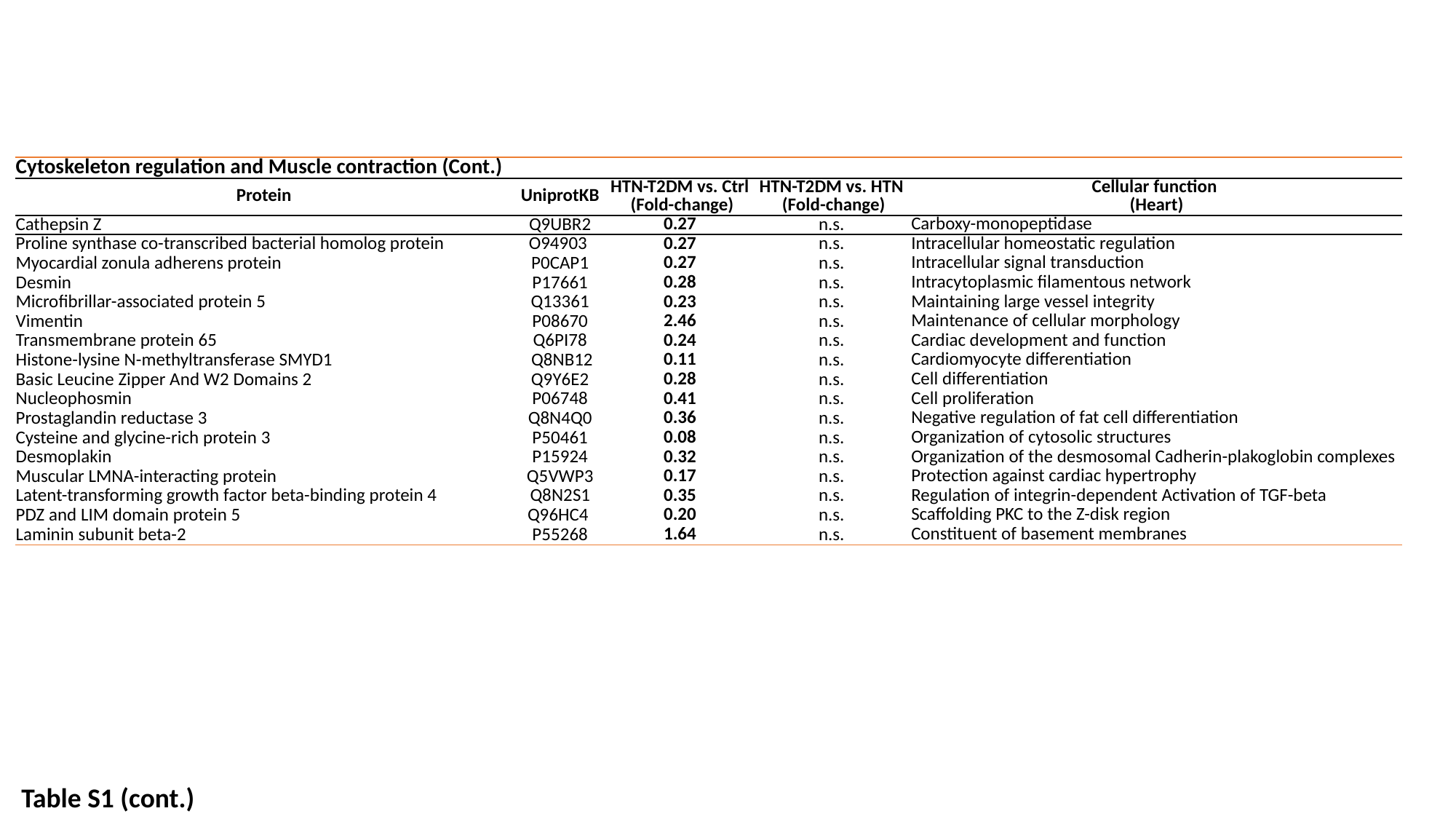

| Cytoskeleton regulation and Muscle contraction (Cont.) | | | | |
| --- | --- | --- | --- | --- |
| Protein | UniprotKB | HTN-T2DM vs. Ctrl (Fold-change) | HTN-T2DM vs. HTN (Fold-change) | Cellular function (Heart) |
| Cathepsin Z | Q9UBR2 | 0.27 | n.s. | Carboxy-monopeptidase |
| Proline synthase co-transcribed bacterial homolog protein | O94903 | 0.27 | n.s. | Intracellular homeostatic regulation |
| Myocardial zonula adherens protein | P0CAP1 | 0.27 | n.s. | Intracellular signal transduction |
| Desmin | P17661 | 0.28 | n.s. | Intracytoplasmic filamentous network |
| Microfibrillar-associated protein 5 | Q13361 | 0.23 | n.s. | Maintaining large vessel integrity |
| Vimentin | P08670 | 2.46 | n.s. | Maintenance of cellular morphology |
| Transmembrane protein 65 | Q6PI78 | 0.24 | n.s. | Cardiac development and function |
| Histone-lysine N-methyltransferase SMYD1 | Q8NB12 | 0.11 | n.s. | Cardiomyocyte differentiation |
| Basic Leucine Zipper And W2 Domains 2 | Q9Y6E2 | 0.28 | n.s. | Cell differentiation |
| Nucleophosmin | P06748 | 0.41 | n.s. | Cell proliferation |
| Prostaglandin reductase 3 | Q8N4Q0 | 0.36 | n.s. | Negative regulation of fat cell differentiation |
| Cysteine and glycine-rich protein 3 | P50461 | 0.08 | n.s. | Organization of cytosolic structures |
| Desmoplakin | P15924 | 0.32 | n.s. | Organization of the desmosomal Cadherin-plakoglobin complexes |
| Muscular LMNA-interacting protein | Q5VWP3 | 0.17 | n.s. | Protection against cardiac hypertrophy |
| Latent-transforming growth factor beta-binding protein 4 | Q8N2S1 | 0.35 | n.s. | Regulation of integrin-dependent Activation of TGF-beta |
| PDZ and LIM domain protein 5 | Q96HC4 | 0.20 | n.s. | Scaffolding PKC to the Z-disk region |
| Laminin subunit beta-2 | P55268 | 1.64 | n.s. | Constituent of basement membranes |
Table S1 (cont.)

## Slide 17
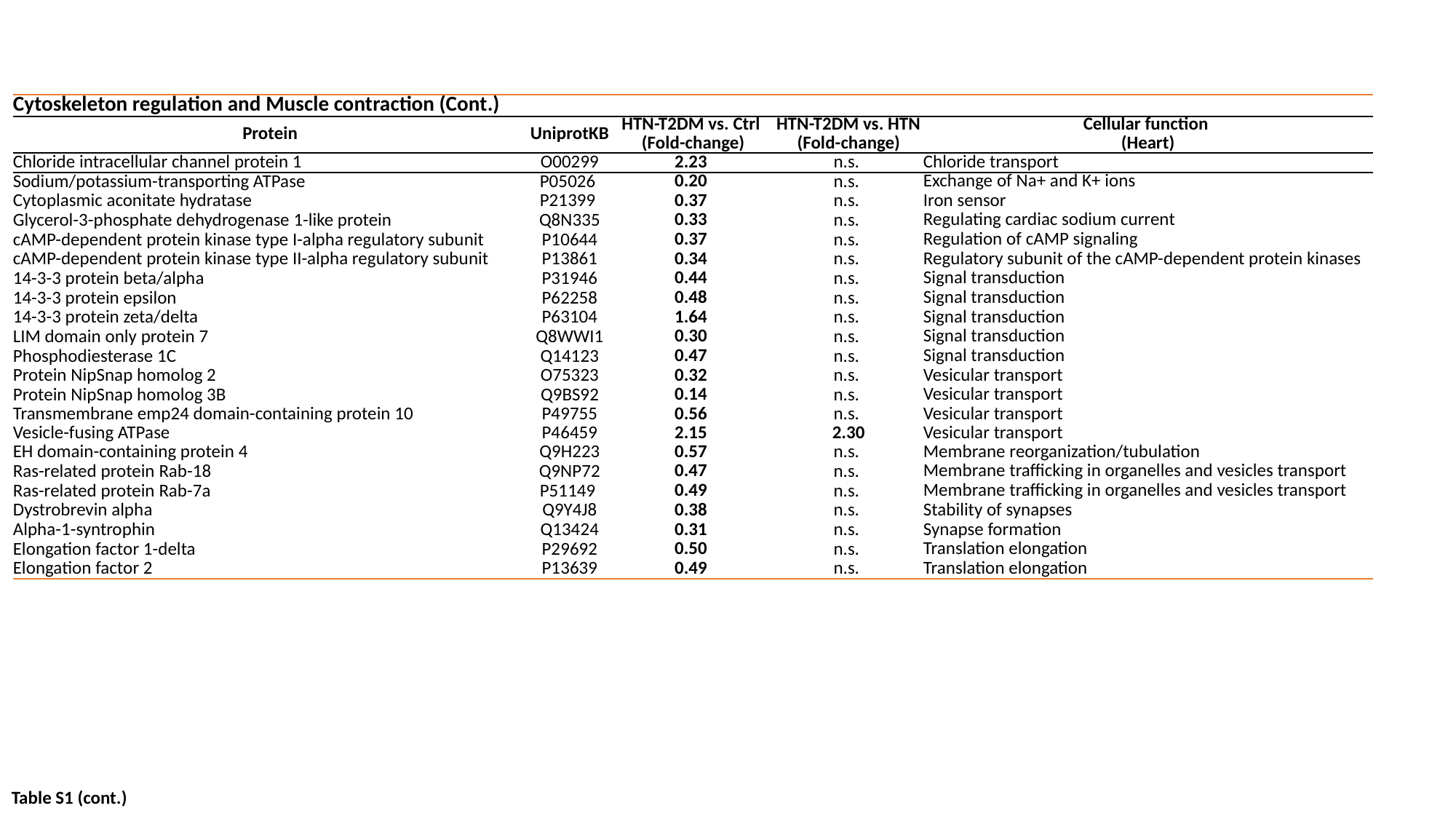

| Cytoskeleton regulation and Muscle contraction (Cont.) | | | | |
| --- | --- | --- | --- | --- |
| Protein | UniprotKB | HTN-T2DM vs. Ctrl (Fold-change) | HTN-T2DM vs. HTN (Fold-change) | Cellular function (Heart) |
| Chloride intracellular channel protein 1 | O00299 | 2.23 | n.s. | Chloride transport |
| Sodium/potassium-transporting ATPase | P05026 | 0.20 | n.s. | Exchange of Na+ and K+ ions |
| Cytoplasmic aconitate hydratase | P21399 | 0.37 | n.s. | Iron sensor |
| Glycerol-3-phosphate dehydrogenase 1-like protein | Q8N335 | 0.33 | n.s. | Regulating cardiac sodium current |
| cAMP-dependent protein kinase type I-alpha regulatory subunit | P10644 | 0.37 | n.s. | Regulation of cAMP signaling |
| cAMP-dependent protein kinase type II-alpha regulatory subunit | P13861 | 0.34 | n.s. | Regulatory subunit of the cAMP-dependent protein kinases |
| 14-3-3 protein beta/alpha | P31946 | 0.44 | n.s. | Signal transduction |
| 14-3-3 protein epsilon | P62258 | 0.48 | n.s. | Signal transduction |
| 14-3-3 protein zeta/delta | P63104 | 1.64 | n.s. | Signal transduction |
| LIM domain only protein 7 | Q8WWI1 | 0.30 | n.s. | Signal transduction |
| Phosphodiesterase 1C | Q14123 | 0.47 | n.s. | Signal transduction |
| Protein NipSnap homolog 2 | O75323 | 0.32 | n.s. | Vesicular transport |
| Protein NipSnap homolog 3B | Q9BS92 | 0.14 | n.s. | Vesicular transport |
| Transmembrane emp24 domain-containing protein 10 | P49755 | 0.56 | n.s. | Vesicular transport |
| Vesicle-fusing ATPase | P46459 | 2.15 | 2.30 | Vesicular transport |
| EH domain-containing protein 4 | Q9H223 | 0.57 | n.s. | Membrane reorganization/tubulation |
| Ras-related protein Rab-18 | Q9NP72 | 0.47 | n.s. | Membrane trafficking in organelles and vesicles transport |
| Ras-related protein Rab-7a | P51149 | 0.49 | n.s. | Membrane trafficking in organelles and vesicles transport |
| Dystrobrevin alpha | Q9Y4J8 | 0.38 | n.s. | Stability of synapses |
| Alpha-1-syntrophin | Q13424 | 0.31 | n.s. | Synapse formation |
| Elongation factor 1-delta | P29692 | 0.50 | n.s. | Translation elongation |
| Elongation factor 2 | P13639 | 0.49 | n.s. | Translation elongation |
Table S1 (cont.)

## Slide 18
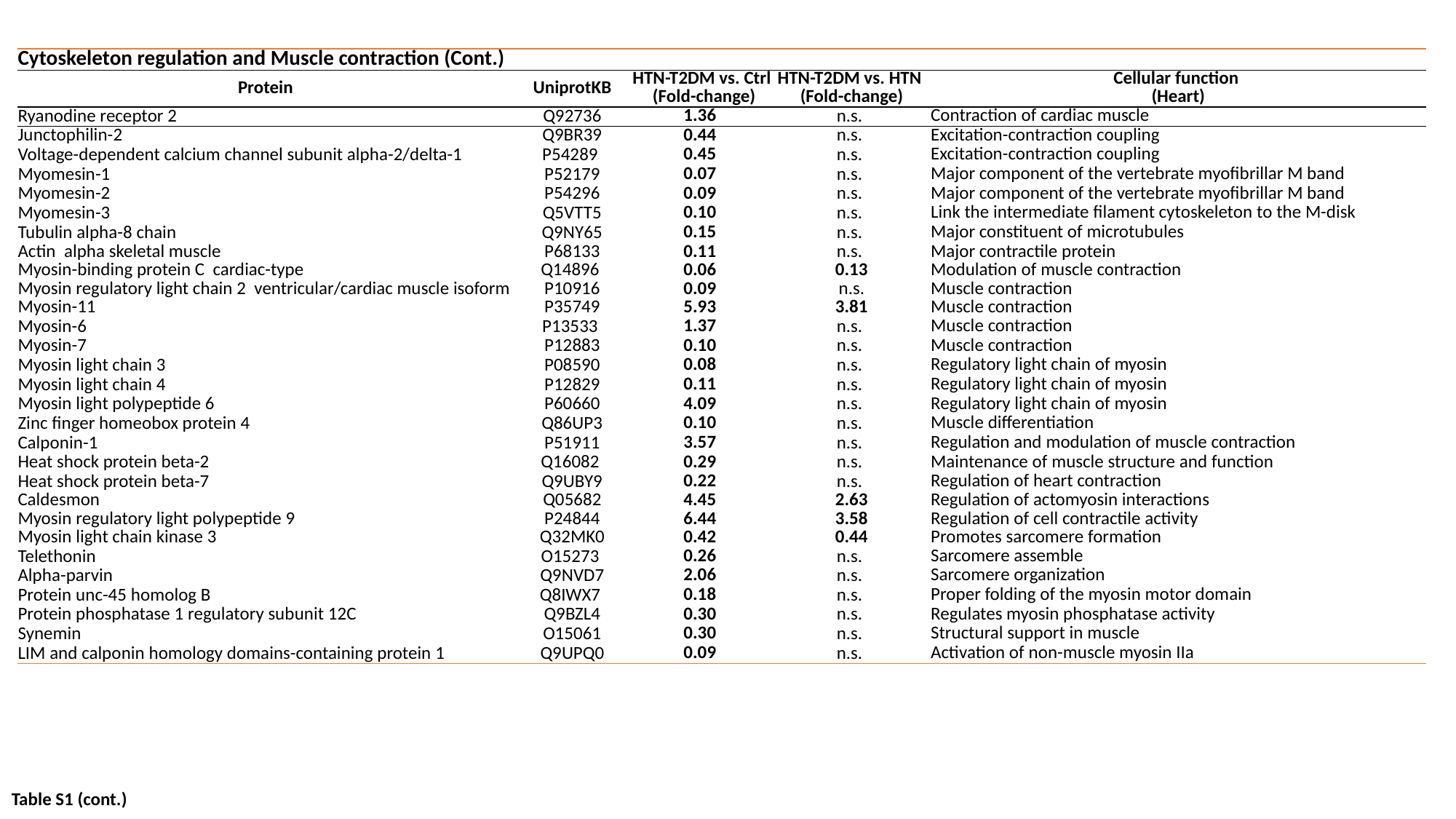

| Cytoskeleton regulation and Muscle contraction (Cont.) | | | | |
| --- | --- | --- | --- | --- |
| Protein | UniprotKB | HTN-T2DM vs. Ctrl (Fold-change) | HTN-T2DM vs. HTN (Fold-change) | Cellular function (Heart) |
| Ryanodine receptor 2 | Q92736 | 1.36 | n.s. | Contraction of cardiac muscle |
| Junctophilin-2 | Q9BR39 | 0.44 | n.s. | Excitation-contraction coupling |
| Voltage-dependent calcium channel subunit alpha-2/delta-1 | P54289 | 0.45 | n.s. | Excitation-contraction coupling |
| Myomesin-1 | P52179 | 0.07 | n.s. | Major component of the vertebrate myofibrillar M band |
| Myomesin-2 | P54296 | 0.09 | n.s. | Major component of the vertebrate myofibrillar M band |
| Myomesin-3 | Q5VTT5 | 0.10 | n.s. | Link the intermediate filament cytoskeleton to the M-disk |
| Tubulin alpha-8 chain | Q9NY65 | 0.15 | n.s. | Major constituent of microtubules |
| Actin alpha skeletal muscle | P68133 | 0.11 | n.s. | Major contractile protein |
| Myosin-binding protein C cardiac-type | Q14896 | 0.06 | 0.13 | Modulation of muscle contraction |
| Myosin regulatory light chain 2 ventricular/cardiac muscle isoform | P10916 | 0.09 | n.s. | Muscle contraction |
| Myosin-11 | P35749 | 5.93 | 3.81 | Muscle contraction |
| Myosin-6 | P13533 | 1.37 | n.s. | Muscle contraction |
| Myosin-7 | P12883 | 0.10 | n.s. | Muscle contraction |
| Myosin light chain 3 | P08590 | 0.08 | n.s. | Regulatory light chain of myosin |
| Myosin light chain 4 | P12829 | 0.11 | n.s. | Regulatory light chain of myosin |
| Myosin light polypeptide 6 | P60660 | 4.09 | n.s. | Regulatory light chain of myosin |
| Zinc finger homeobox protein 4 | Q86UP3 | 0.10 | n.s. | Muscle differentiation |
| Calponin-1 | P51911 | 3.57 | n.s. | Regulation and modulation of muscle contraction |
| Heat shock protein beta-2 | Q16082 | 0.29 | n.s. | Maintenance of muscle structure and function |
| Heat shock protein beta-7 | Q9UBY9 | 0.22 | n.s. | Regulation of heart contraction |
| Caldesmon | Q05682 | 4.45 | 2.63 | Regulation of actomyosin interactions |
| Myosin regulatory light polypeptide 9 | P24844 | 6.44 | 3.58 | Regulation of cell contractile activity |
| Myosin light chain kinase 3 | Q32MK0 | 0.42 | 0.44 | Promotes sarcomere formation |
| Telethonin | O15273 | 0.26 | n.s. | Sarcomere assemble |
| Alpha-parvin | Q9NVD7 | 2.06 | n.s. | Sarcomere organization |
| Protein unc-45 homolog B | Q8IWX7 | 0.18 | n.s. | Proper folding of the myosin motor domain |
| Protein phosphatase 1 regulatory subunit 12C | Q9BZL4 | 0.30 | n.s. | Regulates myosin phosphatase activity |
| Synemin | O15061 | 0.30 | n.s. | Structural support in muscle |
| LIM and calponin homology domains-containing protein 1 | Q9UPQ0 | 0.09 | n.s. | Activation of non-muscle myosin IIa |
Table S1 (cont.)

## Slide 19
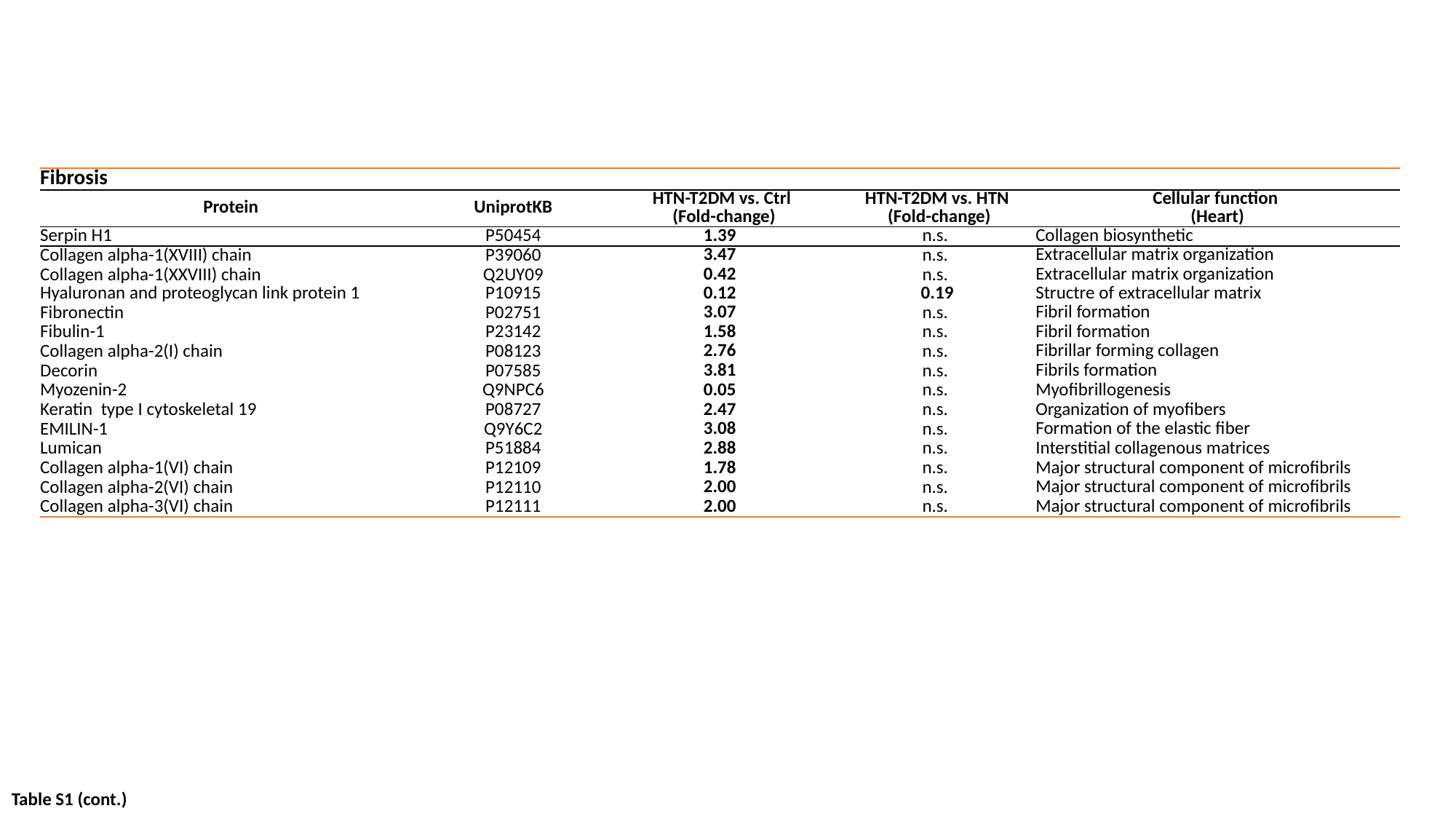

| Fibrosis | | | | |
| --- | --- | --- | --- | --- |
| Protein | UniprotKB | HTN-T2DM vs. Ctrl (Fold-change) | HTN-T2DM vs. HTN (Fold-change) | Cellular function (Heart) |
| Serpin H1 | P50454 | 1.39 | n.s. | Collagen biosynthetic |
| Collagen alpha-1(XVIII) chain | P39060 | 3.47 | n.s. | Extracellular matrix organization |
| Collagen alpha-1(XXVIII) chain | Q2UY09 | 0.42 | n.s. | Extracellular matrix organization |
| Hyaluronan and proteoglycan link protein 1 | P10915 | 0.12 | 0.19 | Structre of extracellular matrix |
| Fibronectin | P02751 | 3.07 | n.s. | Fibril formation |
| Fibulin-1 | P23142 | 1.58 | n.s. | Fibril formation |
| Collagen alpha-2(I) chain | P08123 | 2.76 | n.s. | Fibrillar forming collagen |
| Decorin | P07585 | 3.81 | n.s. | Fibrils formation |
| Myozenin-2 | Q9NPC6 | 0.05 | n.s. | Myofibrillogenesis |
| Keratin type I cytoskeletal 19 | P08727 | 2.47 | n.s. | Organization of myofibers |
| EMILIN-1 | Q9Y6C2 | 3.08 | n.s. | Formation of the elastic fiber |
| Lumican | P51884 | 2.88 | n.s. | Interstitial collagenous matrices |
| Collagen alpha-1(VI) chain | P12109 | 1.78 | n.s. | Major structural component of microfibrils |
| Collagen alpha-2(VI) chain | P12110 | 2.00 | n.s. | Major structural component of microfibrils |
| Collagen alpha-3(VI) chain | P12111 | 2.00 | n.s. | Major structural component of microfibrils |
Table S1 (cont.)

## Slide 20
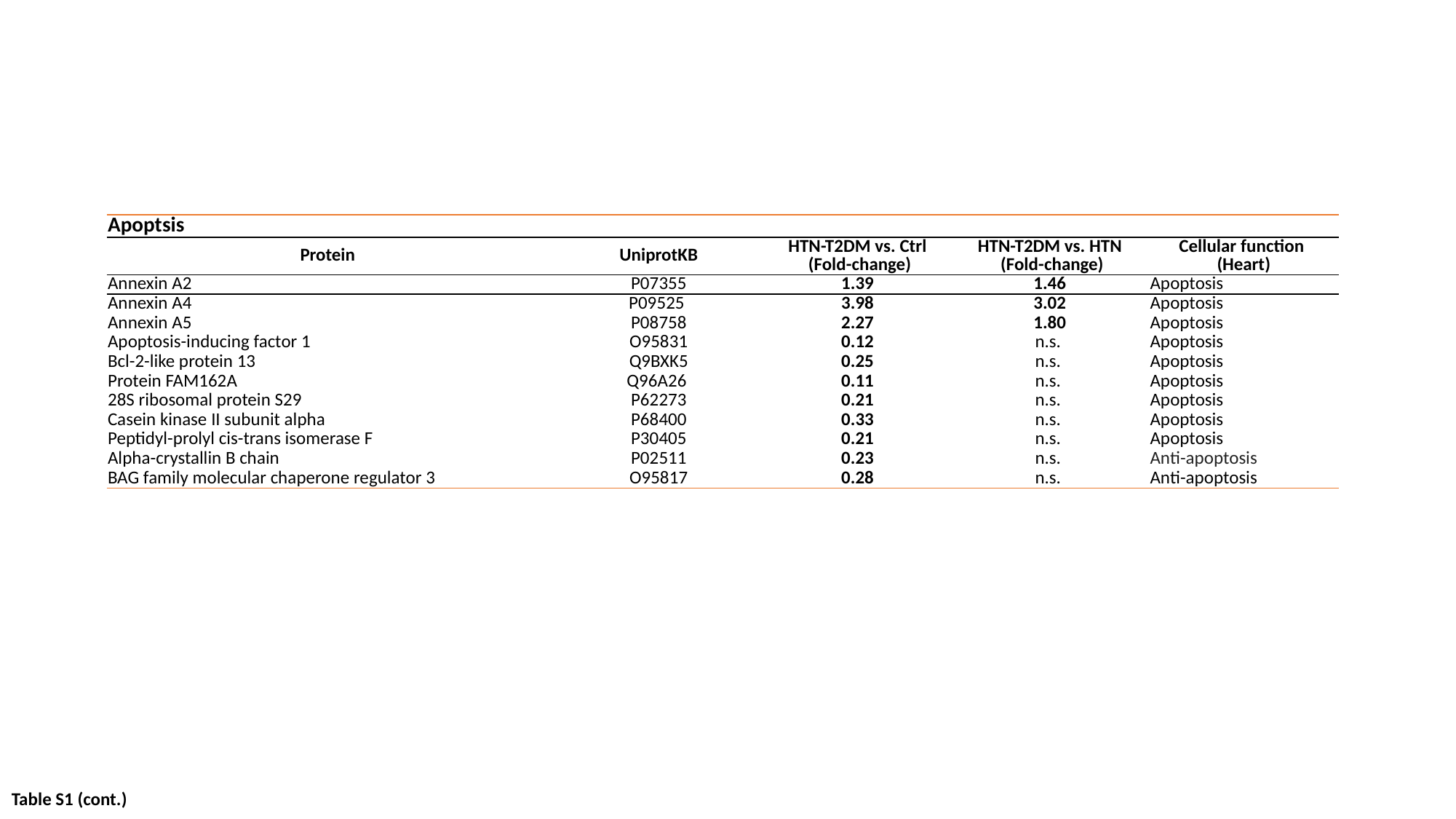

| Apoptsis | | | | |
| --- | --- | --- | --- | --- |
| Protein | UniprotKB | HTN-T2DM vs. Ctrl (Fold-change) | HTN-T2DM vs. HTN (Fold-change) | Cellular function (Heart) |
| Annexin A2 | P07355 | 1.39 | 1.46 | Apoptosis |
| Annexin A4 | P09525 | 3.98 | 3.02 | Apoptosis |
| Annexin A5 | P08758 | 2.27 | 1.80 | Apoptosis |
| Apoptosis-inducing factor 1 | O95831 | 0.12 | n.s. | Apoptosis |
| Bcl-2-like protein 13 | Q9BXK5 | 0.25 | n.s. | Apoptosis |
| Protein FAM162A | Q96A26 | 0.11 | n.s. | Apoptosis |
| 28S ribosomal protein S29 | P62273 | 0.21 | n.s. | Apoptosis |
| Casein kinase II subunit alpha | P68400 | 0.33 | n.s. | Apoptosis |
| Peptidyl-prolyl cis-trans isomerase F | P30405 | 0.21 | n.s. | Apoptosis |
| Alpha-crystallin B chain | P02511 | 0.23 | n.s. | Anti-apoptosis |
| BAG family molecular chaperone regulator 3 | O95817 | 0.28 | n.s. | Anti-apoptosis |
Table S1 (cont.)

## Slide 21
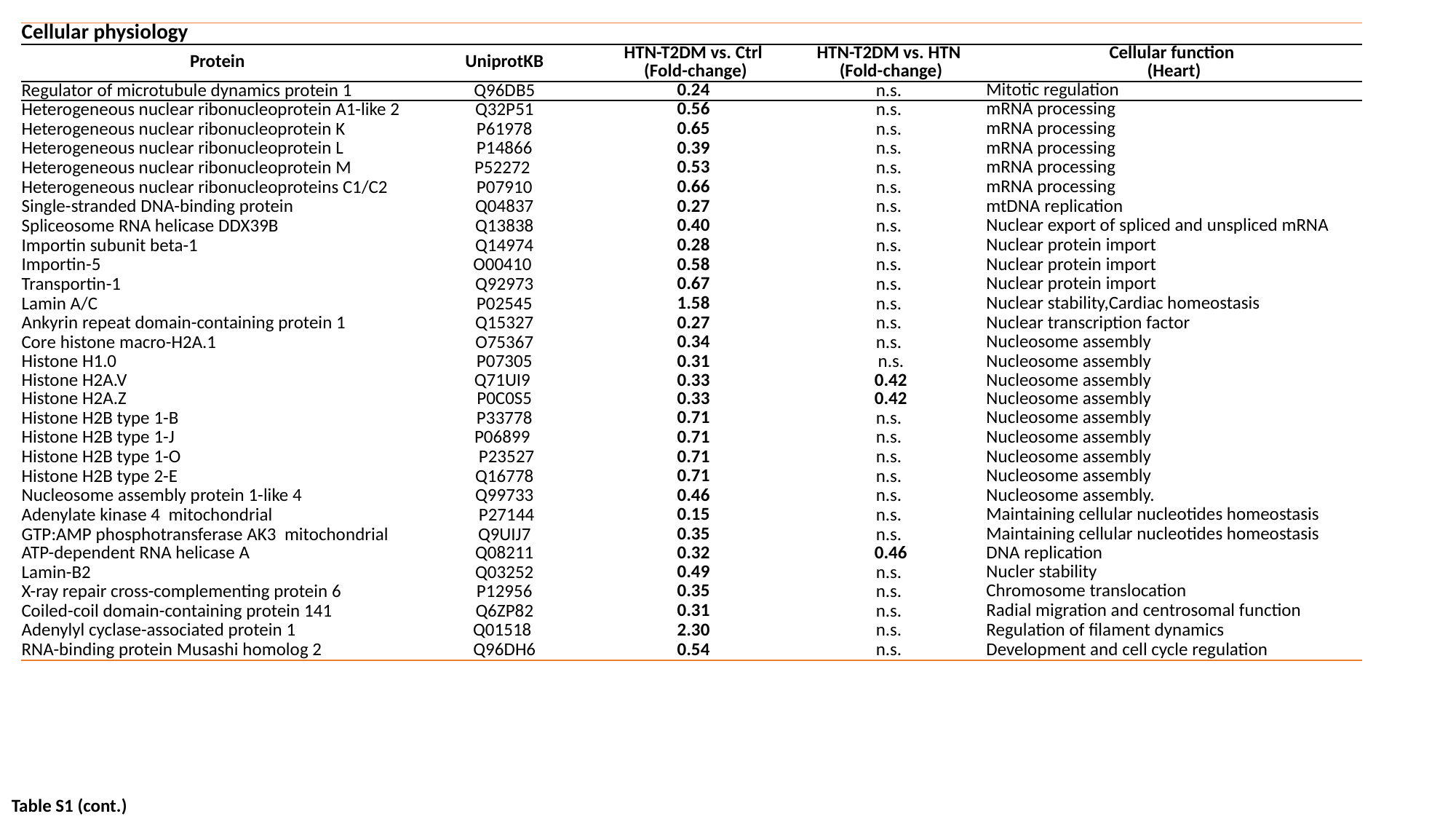

| Cellular physiology | | | | |
| --- | --- | --- | --- | --- |
| Protein | UniprotKB | HTN-T2DM vs. Ctrl (Fold-change) | HTN-T2DM vs. HTN (Fold-change) | Cellular function (Heart) |
| Regulator of microtubule dynamics protein 1 | Q96DB5 | 0.24 | n.s. | Mitotic regulation |
| Heterogeneous nuclear ribonucleoprotein A1-like 2 | Q32P51 | 0.56 | n.s. | mRNA processing |
| Heterogeneous nuclear ribonucleoprotein K | P61978 | 0.65 | n.s. | mRNA processing |
| Heterogeneous nuclear ribonucleoprotein L | P14866 | 0.39 | n.s. | mRNA processing |
| Heterogeneous nuclear ribonucleoprotein M | P52272 | 0.53 | n.s. | mRNA processing |
| Heterogeneous nuclear ribonucleoproteins C1/C2 | P07910 | 0.66 | n.s. | mRNA processing |
| Single-stranded DNA-binding protein | Q04837 | 0.27 | n.s. | mtDNA replication |
| Spliceosome RNA helicase DDX39B | Q13838 | 0.40 | n.s. | Nuclear export of spliced and unspliced mRNA |
| Importin subunit beta-1 | Q14974 | 0.28 | n.s. | Nuclear protein import |
| Importin-5 | O00410 | 0.58 | n.s. | Nuclear protein import |
| Transportin-1 | Q92973 | 0.67 | n.s. | Nuclear protein import |
| Lamin A/C | P02545 | 1.58 | n.s. | Nuclear stability,Cardiac homeostasis |
| Ankyrin repeat domain-containing protein 1 | Q15327 | 0.27 | n.s. | Nuclear transcription factor |
| Core histone macro-H2A.1 | O75367 | 0.34 | n.s. | Nucleosome assembly |
| Histone H1.0 | P07305 | 0.31 | n.s. | Nucleosome assembly |
| Histone H2A.V | Q71UI9 | 0.33 | 0.42 | Nucleosome assembly |
| Histone H2A.Z | P0C0S5 | 0.33 | 0.42 | Nucleosome assembly |
| Histone H2B type 1-B | P33778 | 0.71 | n.s. | Nucleosome assembly |
| Histone H2B type 1-J | P06899 | 0.71 | n.s. | Nucleosome assembly |
| Histone H2B type 1-O | P23527 | 0.71 | n.s. | Nucleosome assembly |
| Histone H2B type 2-E | Q16778 | 0.71 | n.s. | Nucleosome assembly |
| Nucleosome assembly protein 1-like 4 | Q99733 | 0.46 | n.s. | Nucleosome assembly. |
| Adenylate kinase 4 mitochondrial | P27144 | 0.15 | n.s. | Maintaining cellular nucleotides homeostasis |
| GTP:AMP phosphotransferase AK3 mitochondrial | Q9UIJ7 | 0.35 | n.s. | Maintaining cellular nucleotides homeostasis |
| ATP-dependent RNA helicase A | Q08211 | 0.32 | 0.46 | DNA replication |
| Lamin-B2 | Q03252 | 0.49 | n.s. | Nucler stability |
| X-ray repair cross-complementing protein 6 | P12956 | 0.35 | n.s. | Chromosome translocation |
| Coiled-coil domain-containing protein 141 | Q6ZP82 | 0.31 | n.s. | Radial migration and centrosomal function |
| Adenylyl cyclase-associated protein 1 | Q01518 | 2.30 | n.s. | Regulation of filament dynamics |
| RNA-binding protein Musashi homolog 2 | Q96DH6 | 0.54 | n.s. | Development and cell cycle regulation |
Table S1 (cont.)

## Slide 22
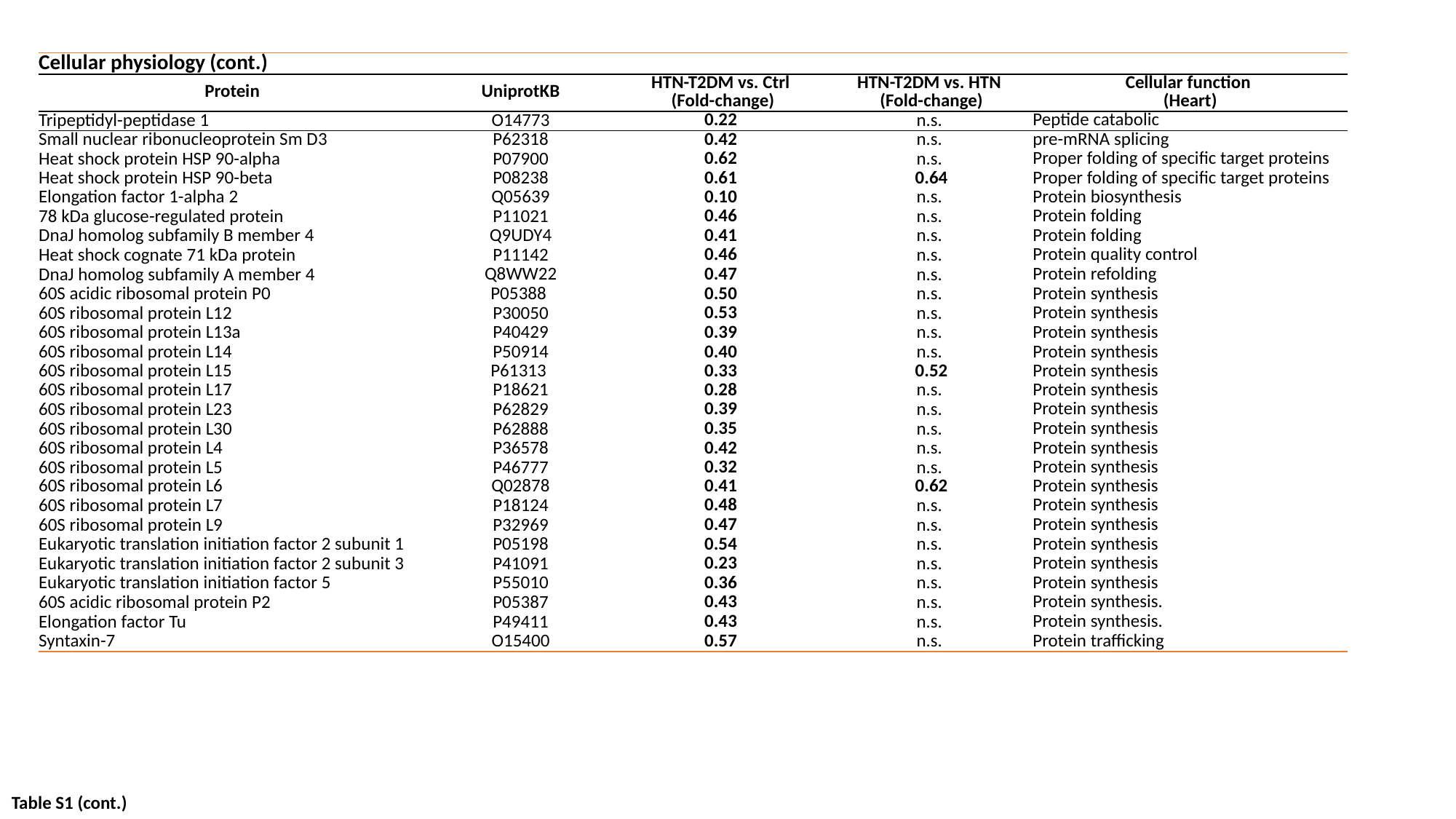

| Cellular physiology (cont.) | | | | |
| --- | --- | --- | --- | --- |
| Protein | UniprotKB | HTN-T2DM vs. Ctrl (Fold-change) | HTN-T2DM vs. HTN (Fold-change) | Cellular function (Heart) |
| Tripeptidyl-peptidase 1 | O14773 | 0.22 | n.s. | Peptide catabolic |
| Small nuclear ribonucleoprotein Sm D3 | P62318 | 0.42 | n.s. | pre-mRNA splicing |
| Heat shock protein HSP 90-alpha | P07900 | 0.62 | n.s. | Proper folding of specific target proteins |
| Heat shock protein HSP 90-beta | P08238 | 0.61 | 0.64 | Proper folding of specific target proteins |
| Elongation factor 1-alpha 2 | Q05639 | 0.10 | n.s. | Protein biosynthesis |
| 78 kDa glucose-regulated protein | P11021 | 0.46 | n.s. | Protein folding |
| DnaJ homolog subfamily B member 4 | Q9UDY4 | 0.41 | n.s. | Protein folding |
| Heat shock cognate 71 kDa protein | P11142 | 0.46 | n.s. | Protein quality control |
| DnaJ homolog subfamily A member 4 | Q8WW22 | 0.47 | n.s. | Protein refolding |
| 60S acidic ribosomal protein P0 | P05388 | 0.50 | n.s. | Protein synthesis |
| 60S ribosomal protein L12 | P30050 | 0.53 | n.s. | Protein synthesis |
| 60S ribosomal protein L13a | P40429 | 0.39 | n.s. | Protein synthesis |
| 60S ribosomal protein L14 | P50914 | 0.40 | n.s. | Protein synthesis |
| 60S ribosomal protein L15 | P61313 | 0.33 | 0.52 | Protein synthesis |
| 60S ribosomal protein L17 | P18621 | 0.28 | n.s. | Protein synthesis |
| 60S ribosomal protein L23 | P62829 | 0.39 | n.s. | Protein synthesis |
| 60S ribosomal protein L30 | P62888 | 0.35 | n.s. | Protein synthesis |
| 60S ribosomal protein L4 | P36578 | 0.42 | n.s. | Protein synthesis |
| 60S ribosomal protein L5 | P46777 | 0.32 | n.s. | Protein synthesis |
| 60S ribosomal protein L6 | Q02878 | 0.41 | 0.62 | Protein synthesis |
| 60S ribosomal protein L7 | P18124 | 0.48 | n.s. | Protein synthesis |
| 60S ribosomal protein L9 | P32969 | 0.47 | n.s. | Protein synthesis |
| Eukaryotic translation initiation factor 2 subunit 1 | P05198 | 0.54 | n.s. | Protein synthesis |
| Eukaryotic translation initiation factor 2 subunit 3 | P41091 | 0.23 | n.s. | Protein synthesis |
| Eukaryotic translation initiation factor 5 | P55010 | 0.36 | n.s. | Protein synthesis |
| 60S acidic ribosomal protein P2 | P05387 | 0.43 | n.s. | Protein synthesis. |
| Elongation factor Tu | P49411 | 0.43 | n.s. | Protein synthesis. |
| Syntaxin-7 | O15400 | 0.57 | n.s. | Protein trafficking |
Table S1 (cont.)

## Slide 23
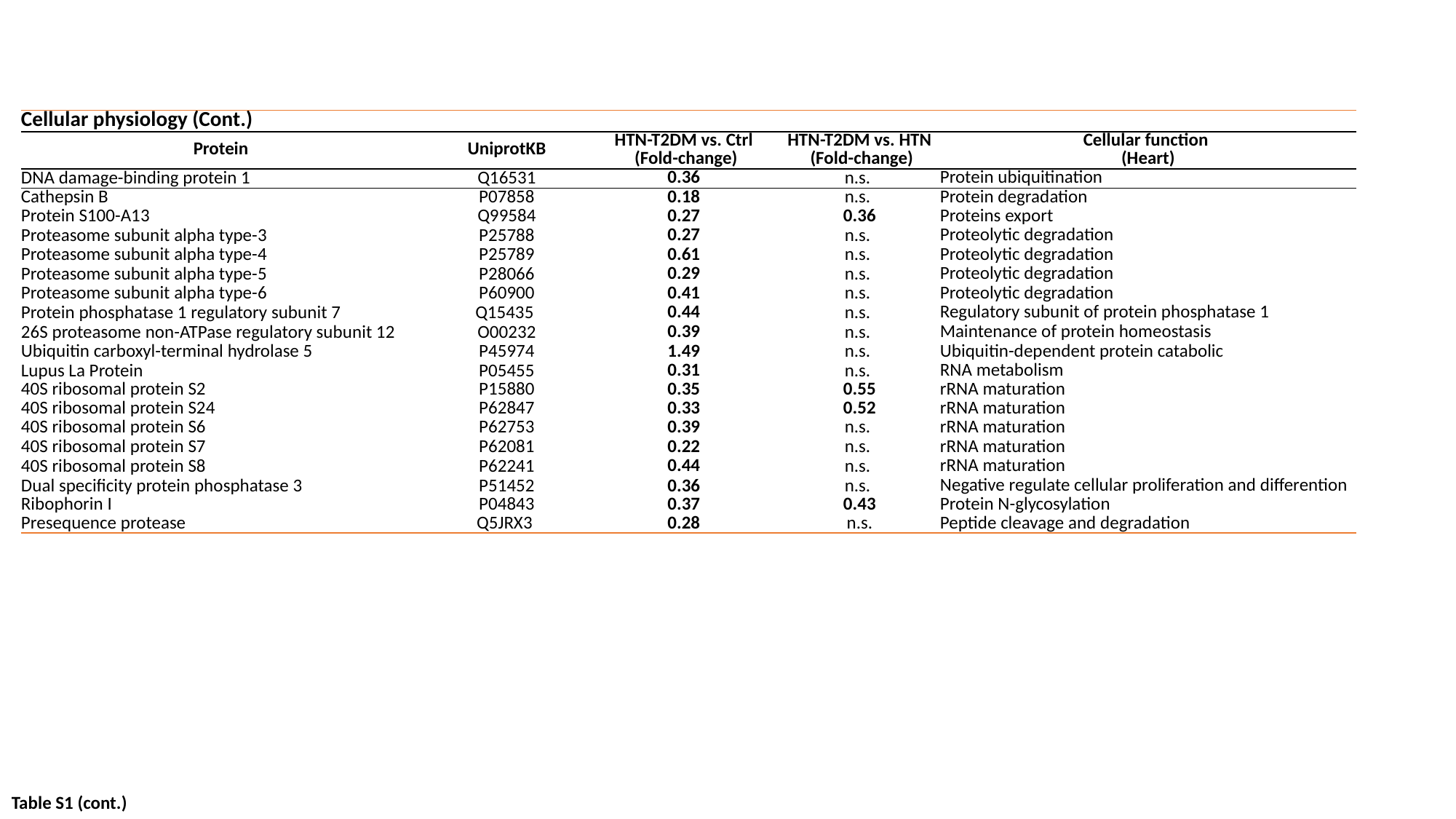

| Cellular physiology (Cont.) | | | | |
| --- | --- | --- | --- | --- |
| Protein | UniprotKB | HTN-T2DM vs. Ctrl (Fold-change) | HTN-T2DM vs. HTN (Fold-change) | Cellular function (Heart) |
| DNA damage-binding protein 1 | Q16531 | 0.36 | n.s. | Protein ubiquitination |
| Cathepsin B | P07858 | 0.18 | n.s. | Protein degradation |
| Protein S100-A13 | Q99584 | 0.27 | 0.36 | Proteins export |
| Proteasome subunit alpha type-3 | P25788 | 0.27 | n.s. | Proteolytic degradation |
| Proteasome subunit alpha type-4 | P25789 | 0.61 | n.s. | Proteolytic degradation |
| Proteasome subunit alpha type-5 | P28066 | 0.29 | n.s. | Proteolytic degradation |
| Proteasome subunit alpha type-6 | P60900 | 0.41 | n.s. | Proteolytic degradation |
| Protein phosphatase 1 regulatory subunit 7 | Q15435 | 0.44 | n.s. | Regulatory subunit of protein phosphatase 1 |
| 26S proteasome non-ATPase regulatory subunit 12 | O00232 | 0.39 | n.s. | Maintenance of protein homeostasis |
| Ubiquitin carboxyl-terminal hydrolase 5 | P45974 | 1.49 | n.s. | Ubiquitin-dependent protein catabolic |
| Lupus La Protein | P05455 | 0.31 | n.s. | RNA metabolism |
| 40S ribosomal protein S2 | P15880 | 0.35 | 0.55 | rRNA maturation |
| 40S ribosomal protein S24 | P62847 | 0.33 | 0.52 | rRNA maturation |
| 40S ribosomal protein S6 | P62753 | 0.39 | n.s. | rRNA maturation |
| 40S ribosomal protein S7 | P62081 | 0.22 | n.s. | rRNA maturation |
| 40S ribosomal protein S8 | P62241 | 0.44 | n.s. | rRNA maturation |
| Dual specificity protein phosphatase 3 | P51452 | 0.36 | n.s. | Negative regulate cellular proliferation and differention |
| Ribophorin I | P04843 | 0.37 | 0.43 | Protein N-glycosylation |
| Presequence protease | Q5JRX3 | 0.28 | n.s. | Peptide cleavage and degradation |
Table S1 (cont.)

## Slide 24
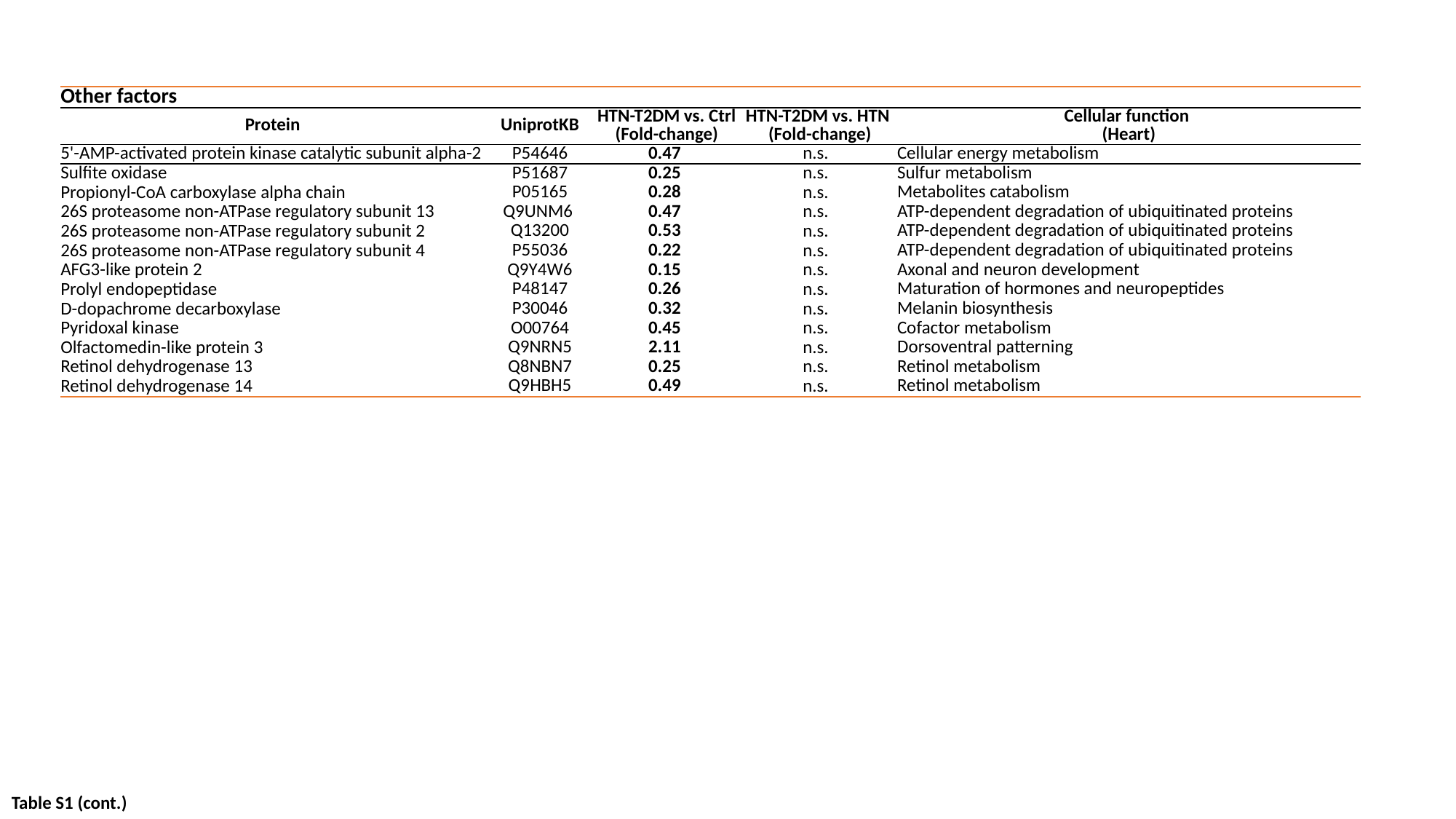

| Other factors | | | | |
| --- | --- | --- | --- | --- |
| Protein | UniprotKB | HTN-T2DM vs. Ctrl (Fold-change) | HTN-T2DM vs. HTN (Fold-change) | Cellular function (Heart) |
| 5'-AMP-activated protein kinase catalytic subunit alpha-2 | P54646 | 0.47 | n.s. | Cellular energy metabolism |
| Sulfite oxidase | P51687 | 0.25 | n.s. | Sulfur metabolism |
| Propionyl-CoA carboxylase alpha chain | P05165 | 0.28 | n.s. | Metabolites catabolism |
| 26S proteasome non-ATPase regulatory subunit 13 | Q9UNM6 | 0.47 | n.s. | ATP-dependent degradation of ubiquitinated proteins |
| 26S proteasome non-ATPase regulatory subunit 2 | Q13200 | 0.53 | n.s. | ATP-dependent degradation of ubiquitinated proteins |
| 26S proteasome non-ATPase regulatory subunit 4 | P55036 | 0.22 | n.s. | ATP-dependent degradation of ubiquitinated proteins |
| AFG3-like protein 2 | Q9Y4W6 | 0.15 | n.s. | Axonal and neuron development |
| Prolyl endopeptidase | P48147 | 0.26 | n.s. | Maturation of hormones and neuropeptides |
| D-dopachrome decarboxylase | P30046 | 0.32 | n.s. | Melanin biosynthesis |
| Pyridoxal kinase | O00764 | 0.45 | n.s. | Cofactor metabolism |
| Olfactomedin-like protein 3 | Q9NRN5 | 2.11 | n.s. | Dorsoventral patterning |
| Retinol dehydrogenase 13 | Q8NBN7 | 0.25 | n.s. | Retinol metabolism |
| Retinol dehydrogenase 14 | Q9HBH5 | 0.49 | n.s. | Retinol metabolism |
Table S1 (cont.)

## Slide 25
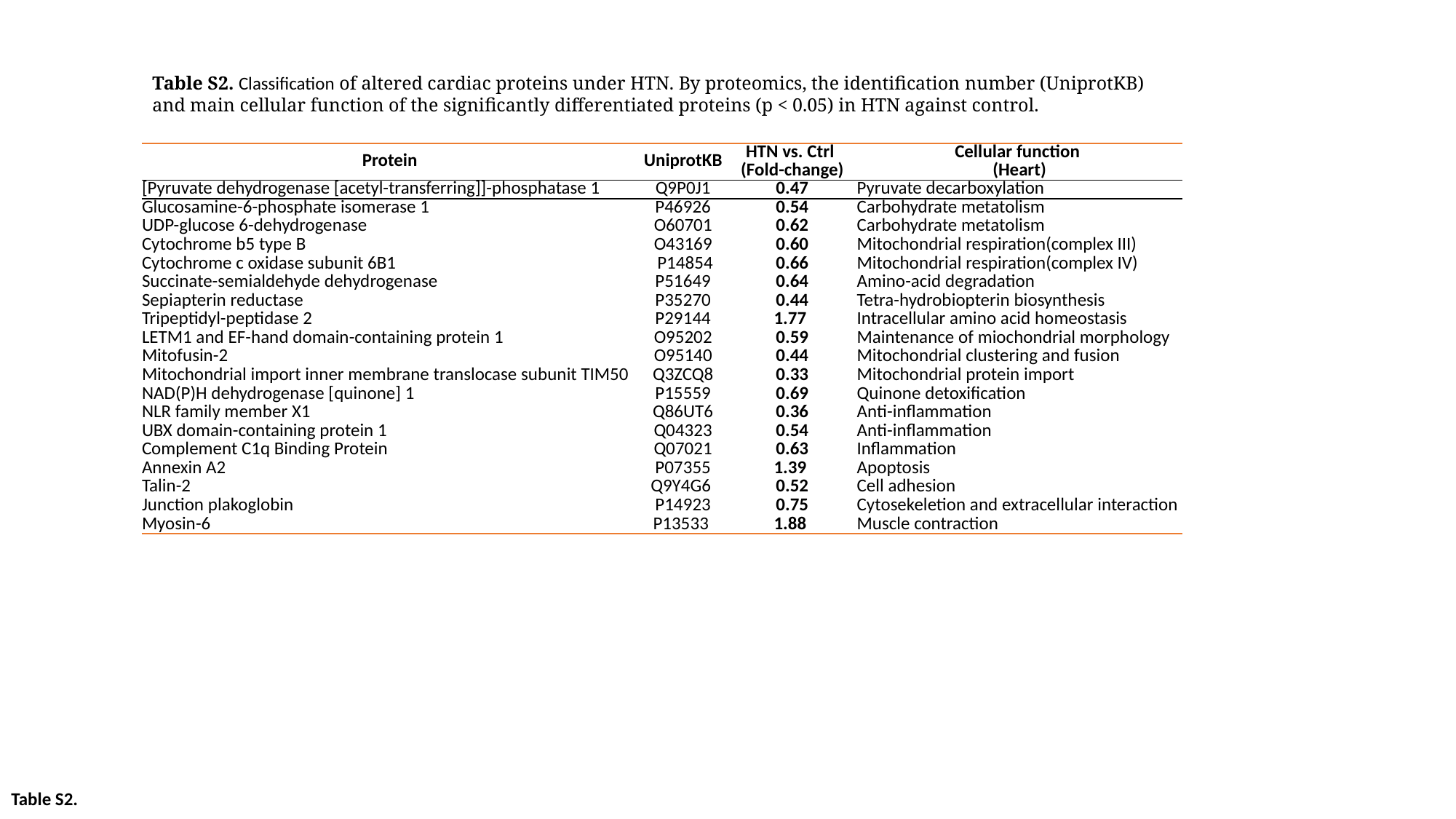

Table S2. Classification of altered cardiac proteins under HTN. By proteomics, the identification number (UniprotKB) and main cellular function of the significantly differentiated proteins (p < 0.05) in HTN against control.
| Protein | UniprotKB | HTN vs. Ctrl (Fold-change) | Cellular function (Heart) |
| --- | --- | --- | --- |
| [Pyruvate dehydrogenase [acetyl-transferring]]-phosphatase 1 | Q9P0J1 | 0.47 | Pyruvate decarboxylation |
| Glucosamine-6-phosphate isomerase 1 | P46926 | 0.54 | Carbohydrate metatolism |
| UDP-glucose 6-dehydrogenase | O60701 | 0.62 | Carbohydrate metatolism |
| Cytochrome b5 type B | O43169 | 0.60 | Mitochondrial respiration(complex III) |
| Cytochrome c oxidase subunit 6B1 | P14854 | 0.66 | Mitochondrial respiration(complex IV) |
| Succinate-semialdehyde dehydrogenase | P51649 | 0.64 | Amino-acid degradation |
| Sepiapterin reductase | P35270 | 0.44 | Tetra-hydrobiopterin biosynthesis |
| Tripeptidyl-peptidase 2 | P29144 | 1.77 | Intracellular amino acid homeostasis |
| LETM1 and EF-hand domain-containing protein 1 | O95202 | 0.59 | Maintenance of miochondrial morphology |
| Mitofusin-2 | O95140 | 0.44 | Mitochondrial clustering and fusion |
| Mitochondrial import inner membrane translocase subunit TIM50 | Q3ZCQ8 | 0.33 | Mitochondrial protein import |
| NAD(P)H dehydrogenase [quinone] 1 | P15559 | 0.69 | Quinone detoxification |
| NLR family member X1 | Q86UT6 | 0.36 | Anti-inflammation |
| UBX domain-containing protein 1 | Q04323 | 0.54 | Anti-inflammation |
| Complement C1q Binding Protein | Q07021 | 0.63 | Inflammation |
| Annexin A2 | P07355 | 1.39 | Apoptosis |
| Talin-2 | Q9Y4G6 | 0.52 | Cell adhesion |
| Junction plakoglobin | P14923 | 0.75 | Cytosekeletion and extracellular interaction |
| Myosin-6 | P13533 | 1.88 | Muscle contraction |
Table S2.

## Slide 26
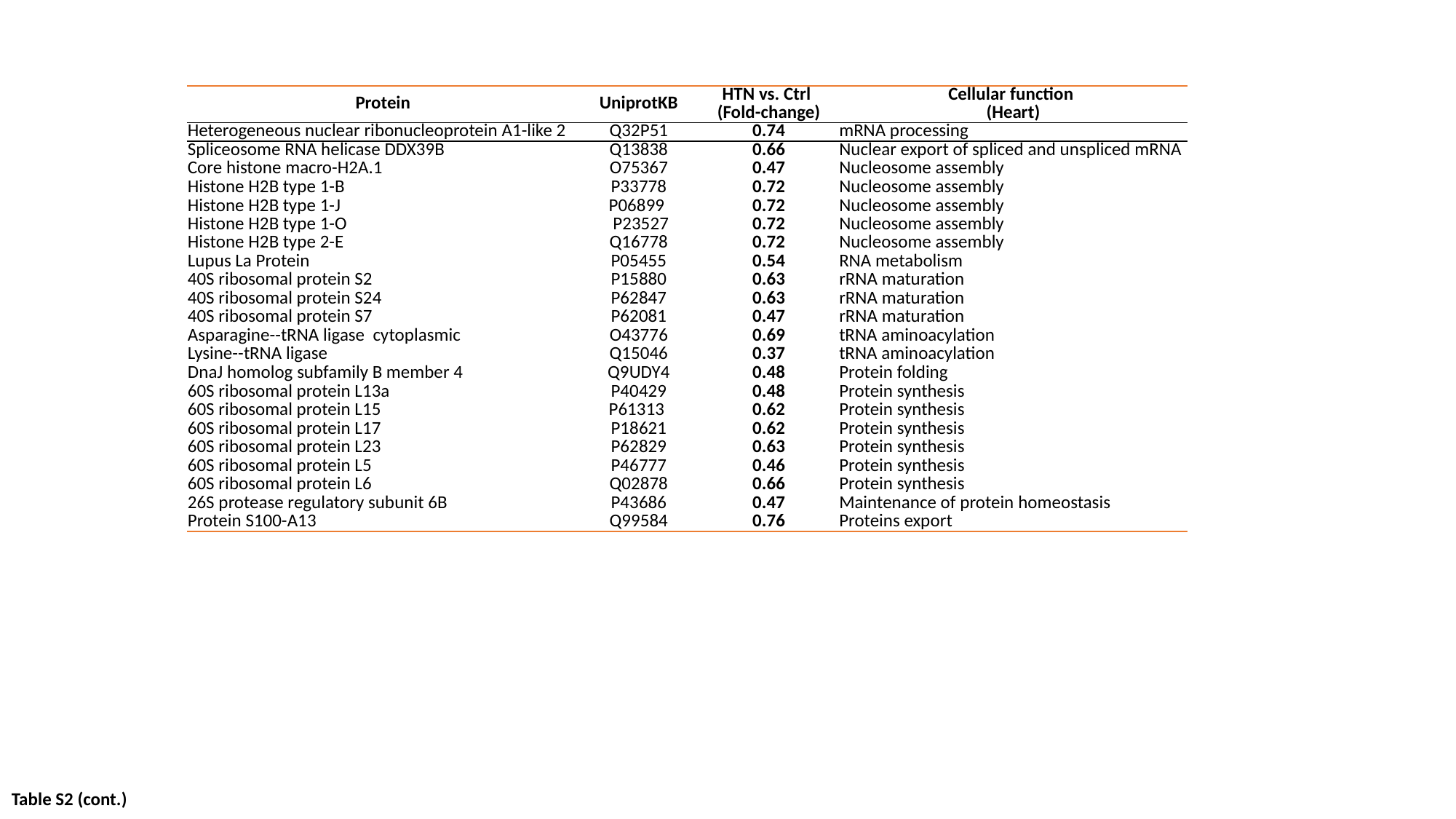

| Protein | UniprotKB | HTN vs. Ctrl (Fold-change) | Cellular function (Heart) |
| --- | --- | --- | --- |
| Heterogeneous nuclear ribonucleoprotein A1-like 2 | Q32P51 | 0.74 | mRNA processing |
| Spliceosome RNA helicase DDX39B | Q13838 | 0.66 | Nuclear export of spliced and unspliced mRNA |
| Core histone macro-H2A.1 | O75367 | 0.47 | Nucleosome assembly |
| Histone H2B type 1-B | P33778 | 0.72 | Nucleosome assembly |
| Histone H2B type 1-J | P06899 | 0.72 | Nucleosome assembly |
| Histone H2B type 1-O | P23527 | 0.72 | Nucleosome assembly |
| Histone H2B type 2-E | Q16778 | 0.72 | Nucleosome assembly |
| Lupus La Protein | P05455 | 0.54 | RNA metabolism |
| 40S ribosomal protein S2 | P15880 | 0.63 | rRNA maturation |
| 40S ribosomal protein S24 | P62847 | 0.63 | rRNA maturation |
| 40S ribosomal protein S7 | P62081 | 0.47 | rRNA maturation |
| Asparagine--tRNA ligase cytoplasmic | O43776 | 0.69 | tRNA aminoacylation |
| Lysine--tRNA ligase | Q15046 | 0.37 | tRNA aminoacylation |
| DnaJ homolog subfamily B member 4 | Q9UDY4 | 0.48 | Protein folding |
| 60S ribosomal protein L13a | P40429 | 0.48 | Protein synthesis |
| 60S ribosomal protein L15 | P61313 | 0.62 | Protein synthesis |
| 60S ribosomal protein L17 | P18621 | 0.62 | Protein synthesis |
| 60S ribosomal protein L23 | P62829 | 0.63 | Protein synthesis |
| 60S ribosomal protein L5 | P46777 | 0.46 | Protein synthesis |
| 60S ribosomal protein L6 | Q02878 | 0.66 | Protein synthesis |
| 26S protease regulatory subunit 6B | P43686 | 0.47 | Maintenance of protein homeostasis |
| Protein S100-A13 | Q99584 | 0.76 | Proteins export |
Table S2 (cont.)

## Slide 27
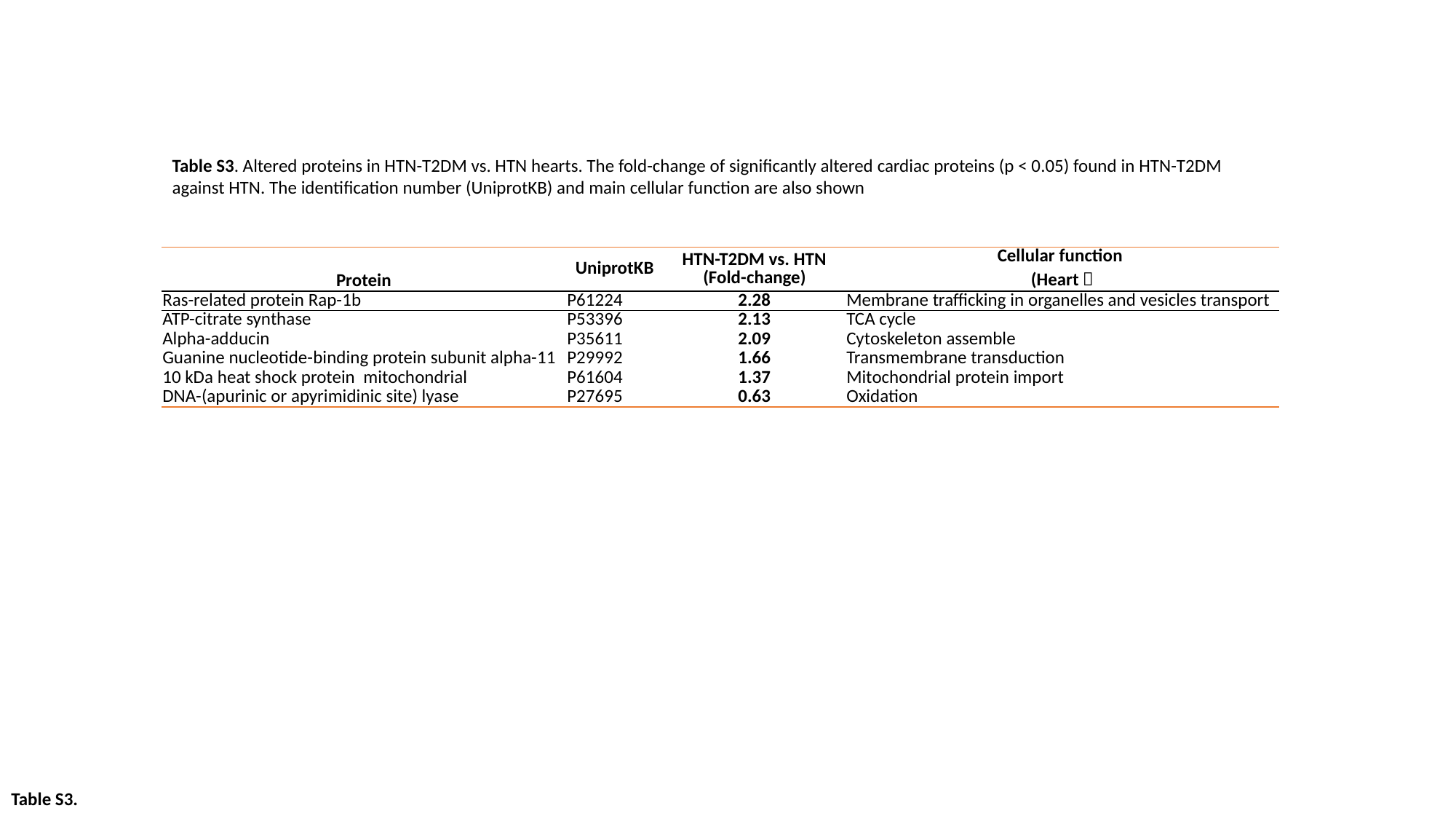

Table S3. Altered proteins in HTN-T2DM vs. HTN hearts. The fold-change of significantly altered cardiac proteins (p < 0.05) found in HTN-T2DM against HTN. The identification number (UniprotKB) and main cellular function are also shown
| Protein | UniprotKB | HTN-T2DM vs. HTN (Fold-change) | Cellular function (Heart） |
| --- | --- | --- | --- |
| Ras-related protein Rap-1b | P61224 | 2.28 | Membrane trafficking in organelles and vesicles transport |
| ATP-citrate synthase | P53396 | 2.13 | TCA cycle |
| Alpha-adducin | P35611 | 2.09 | Cytoskeleton assemble |
| Guanine nucleotide-binding protein subunit alpha-11 | P29992 | 1.66 | Transmembrane transduction |
| 10 kDa heat shock protein mitochondrial | P61604 | 1.37 | Mitochondrial protein import |
| DNA-(apurinic or apyrimidinic site) lyase | P27695 | 0.63 | Oxidation |
Table S3.

## Slide 28
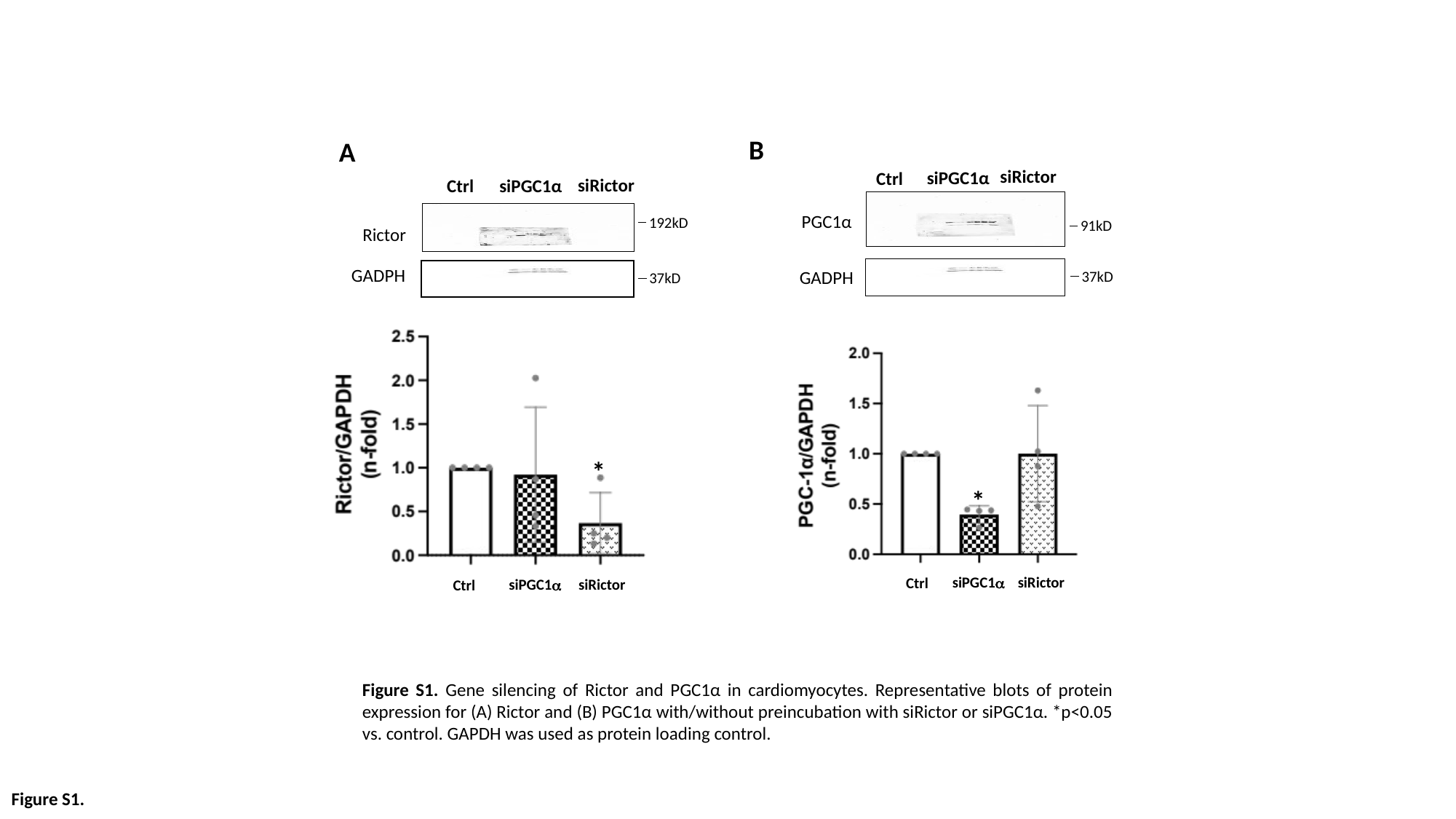

B
siRictor
siPGC1α
Ctrl
PGC1α
GADPH
91kD
37kD
*
siRictor
siPGC1a
Ctrl
A
siRictor
siPGC1α
Ctrl
192kD
Rictor
GADPH
37kD
*
siRictor
siPGC1a
Ctrl
Figure S1. Gene silencing of Rictor and PGC1α in cardiomyocytes. Representative blots of protein expression for (A) Rictor and (B) PGC1α with/without preincubation with siRictor or siPGC1α. *p<0.05 vs. control. GAPDH was used as protein loading control.
Figure S1.

## Slide 29
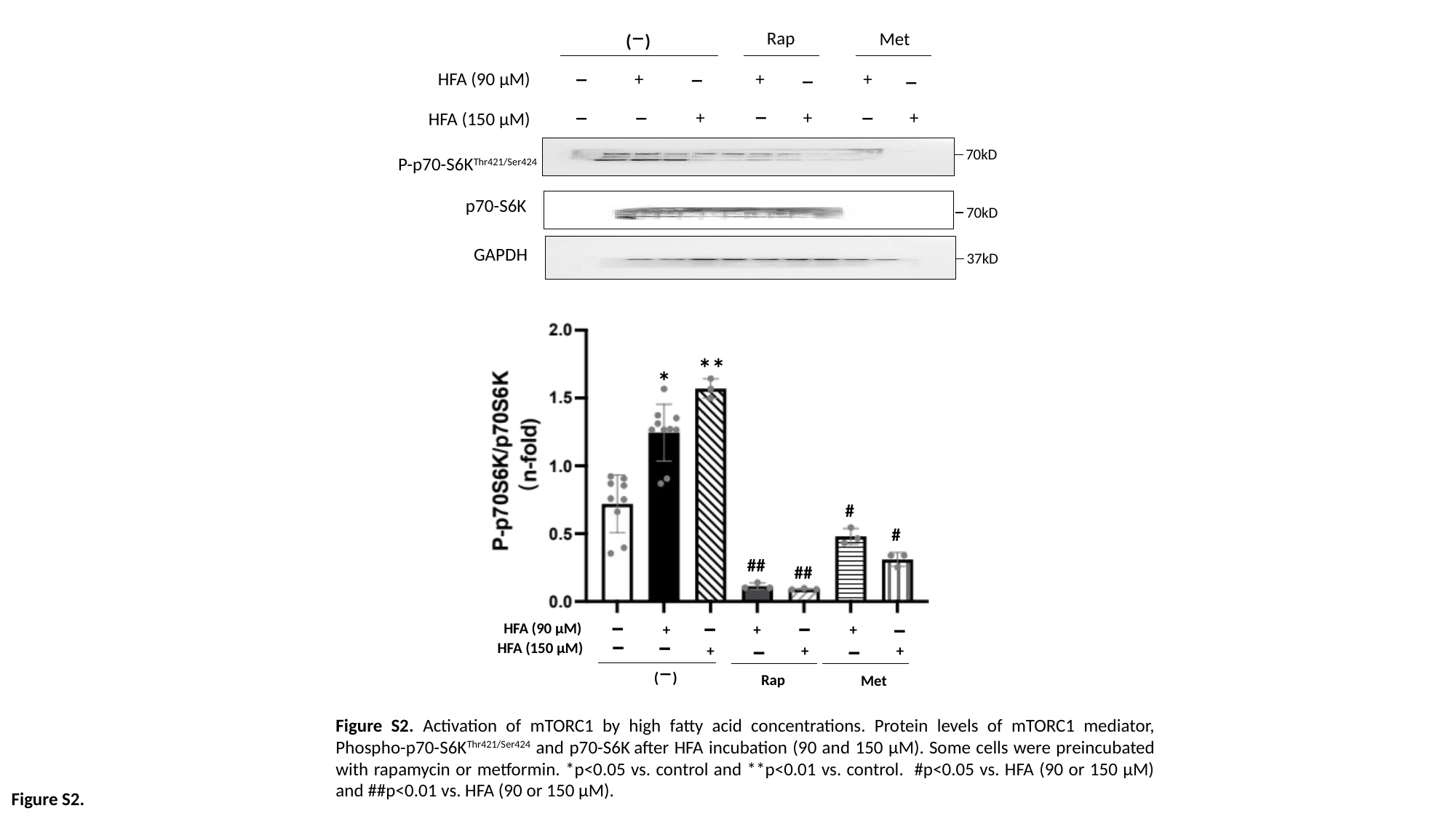

(−)
Rap
Met
−
−
−
−
HFA (90 µM)
+
+
+
−
−
−
−
+
+
+
HFA (150 µM)
70kD
P-p70-S6KThr421/Ser424
GAPDH
p70-S6K
70kD
37kD
**
*
#
#
##
##
−
−
+
−
+
−
−
+
−
+
HFA (90 µM)
+
−
−
HFA (150 µM)
+
(−)
Rap
Met
Figure S2. Activation of mTORC1 by high fatty acid concentrations. Protein levels of mTORC1 mediator, Phospho-p70-S6KThr421/Ser424 and p70-S6K after HFA incubation (90 and 150 μM). Some cells were preincubated with rapamycin or metformin. *p<0.05 vs. control and **p<0.01 vs. control. #p<0.05 vs. HFA (90 or 150 μM) and ##p<0.01 vs. HFA (90 or 150 μM).
Figure S2.

## Slide 30
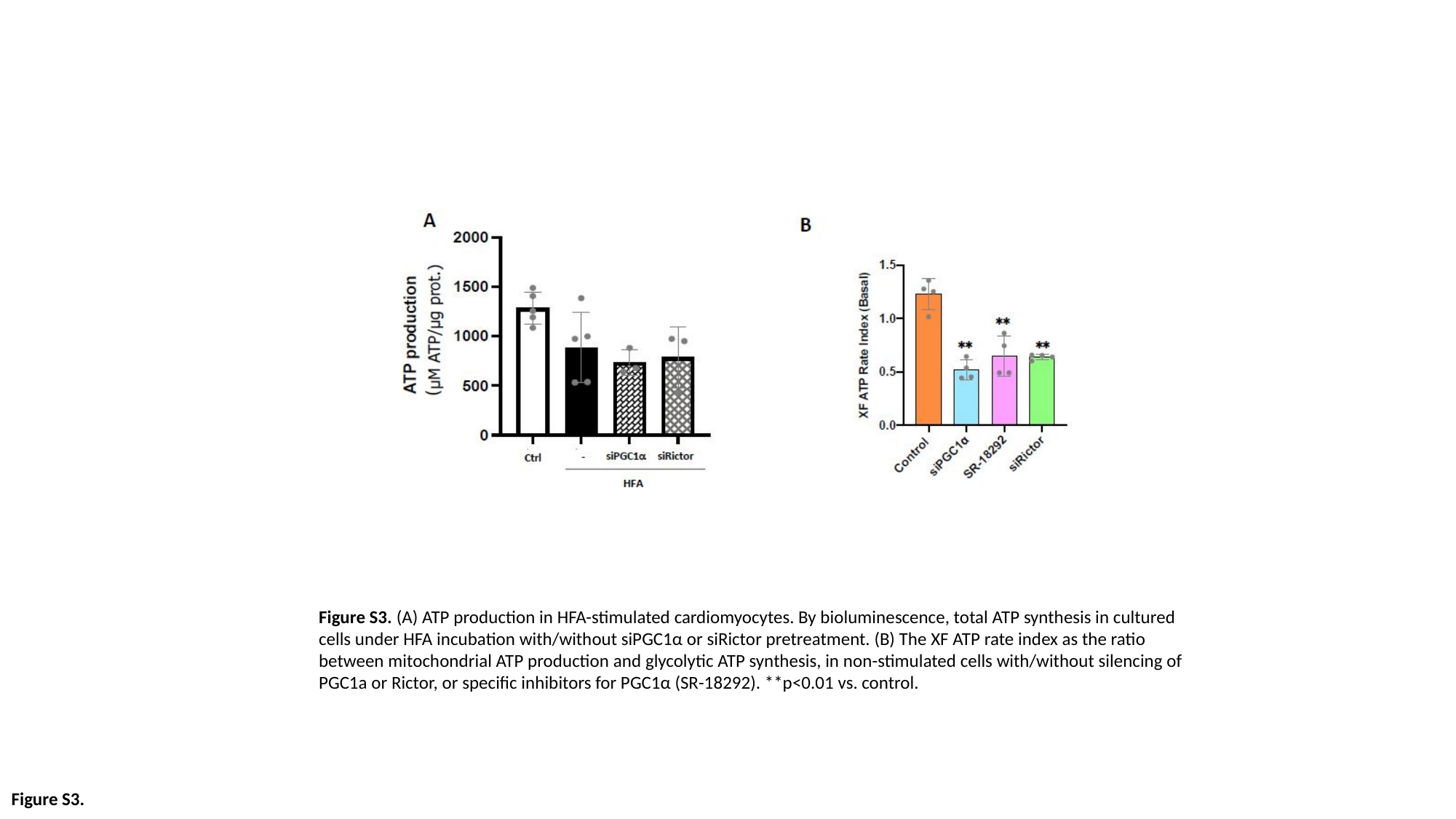

Figure S3. (A) ATP production in HFA-stimulated cardiomyocytes. By bioluminescence, total ATP synthesis in cultured cells under HFA incubation with/without siPGC1α or siRictor pretreatment. (B) The XF ATP rate index as the ratio between mitochondrial ATP production and glycolytic ATP synthesis, in non-stimulated cells with/without silencing of PGC1a or Rictor, or specific inhibitors for PGC1α (SR-18292). **p<0.01 vs. control.
Figure S3.
